# Brain transcriptomic signatures for mood disorders and suicide phenotypes: an anterior insula and subgenual ACC network postmortem study

**DOI:** 10.1016/j.bbih.2025.101051

**Published:** 2025-07-05

**Authors:** Dhivya Arasappan, Abigail Spears, Simran Shah, Roy D. Mayfield, Nirmala Akula, Francis J. McMahon, Mbemba Jabbi

**Affiliations:** a Center for Biomedical Research Support, The University of Texas at Austin, Dell Medical School, Austin, TX, USA; b Department of Psychiatry and Behavioral Sciences, The University of Texas at Austin, Dell Medical School, Austin, TX, USA; c Department of Neuroscience and Waggoner Center for Addiction Research, The University of Texas at Austin, USA; d Genetic Basis of Mood & Anxiety Section, Intramural Research Program, NIMH, NIH, Bethesda, MD, USA; e Center for Learning and Memory, The University of Texas at Austin, Dell Medical School, Austin, TX, USA; f Mulva Clinics for the Neurosciences, Dell Medical School, Austin, TX, USA

## Abstract

Mood disorders affect over ten percent of humans worldwide. Still, studies dissecting the anatomically localized molecular neurobiological mechanisms underlying mood (dys)functions have not consistently identified the patterns of pathological changes in relevant brain regions. Recent studies have identified pathological changes in the anterior insula (Ant-Ins) and subgenual anterior cingulate (sgACC) brain network in mood disorders, in line with this network's role in regulating mood/affective feeling states. Here, we applied whole-tissue RNA-sequencing measures of differentially expressed genes (DEGs) in mood disorders versus (vs.) psychiatrically unaffected controls (controls) to identify postmortem molecular pathological markers for mood disorder phenotypes. Using data reduction/factor analytics of the postmortem phenotypic variables to determine relevant sources of population variances, we identified DEGs associated with mood disorder-related diagnostic phenotypes by combining gene co-expression, differential gene expression, and pathway-enrichment analyses. We found downregulation/underexpression of inflammatory and protein synthesis-related genes associated with increased psychopathological comorbidity (here referred to as *psychiatric morbidity*/a measure of all co-occurring mental disorders and death by suicide) in Ant-Ins, in contrast to upregulation of synaptic membrane and ion channel-related genes with increased *psychiatric morbidity in* sgACC. Our results identified a preponderance of downregulated metabolic, protein synthesis, inflammatory, and synaptic membrane DEGs associated with *suicide* outcomes, and in relation to a factor representing *longevity* in the Ant-Ins and sgACC (AIAC) network. Our study revealed a critical brain network molecular repertoire for mood disorder phenotypes, including suicide outcomes and *longevity*, and provides a framework for defining dosage-sensitive (i.e., downregulated vs. upregulated) molecular signatures for mood phenotypic complexity and pathological consequences.

## 1 Introduction

Major depressive Disorder ‘MDD’ and bipolar disorder ‘BD, including both type I and type II’, together referred to here as mood disorders, are profoundly debilitating brain and behavioral disorders that globally affect about 400 million people annually. Mood disorders inflict a substantial disease burden, cause a significant proportion of the over 800 thousand premature mortalities due to suicide, and are associated with increased adverse socioeconomic consequences and social isolation on the global population (Kessler et al., 2005; Murray et al., 2012). The cumulative co-occurrence of mood disorders and comorbid psychiatric and chronic medical conditions like cardiovascular diseases can exert a compounding negative toll on human well-being, life expectancy, and mortality outcomes (Kessler et al., 2005; Oquendo et al., 2010; Whiteford et al., 2013; Niculescu et al., 2017; Turecki et al., 2019). Although previous research has identified pathobiological markers for prevalent conditions like cardiovascular diseases (Gibbs et al., 2001), which often co-occur with mood disorders, the molecular neurobiological mechanisms underlying mood disorders and comorbid conditions remain unclear.

Of relevance to the behavioral ability to regulate mood functions in health and diseases, the anterior insula cortex (Ant-Ins) and subgenual anterior cingulate cortex (sgACC) brain network is well-documented to harbor the most connections with other brain regions via cortical and sub-cortical telencephalic brain connective fibers (Mesulam, 1998), and with peripheral cardiovascular, gut, and adrenal systems through intricate descending extratelencephalic fibers (Dum et al., 2019; Levinthal and Strick, 2020). The anatomical and functional integrity of the Ant-Ins subregion is critical in engendering *interoceptive* sensing of internal feeling states like pain, itch, taste, smell, body temperature, experience of sickness, and affect/mood (Jabbi et al., 2008; Craig, 2009; Khalsa et al., 2018). Furthermore, the sgACC and adjacent medial prefrontal cortex subregion translate *interoceptive to exteroceptive* sensory domains by integrating external percepts/stimuli like visual and chemosensory cues, with bodily feeling states, to engender subjective emotional and mood tones/feelings (Nauta, 1972; Goldman-Rakic, 1988; Harrison et al., 2009; Joyce and Barbas, 2018; Wang et al., 2021). Together, the anterior insula-subgenual cingulate cortical brain network (we refer to the studied brain network hereafter as AIAC) integrates incoming stimuli with resulting feeling states induced by those stimuli or the imagination of those stimuli (Jabbi et al., 2008) and is hypothesized to regulate mood tone (Drevets et al., 2008).

The AIAC network is well-documented to contribute critical anatomical and physiological involvements in mediating bodily feeling states and affective/mood functions (Wang et al., 2021), as well as in coding and regulation/toning of the related physical sensations and abstract meaning of bodily and affective/mood states in the form of experiential, imagined, and social/empathic processes (Jabbi et al., 2008). Furthermore, the AIAC network's anatomical connection with peripheral systems such as the gut, heart, and adrenal glands, coupled with this brain network's documented anatomical and physiological alterations (e.g., reduced gray matter integrity) in mood and related behavioral and cognitive disorders (Goodkind et al., 2015; Wise et al., 2017; Jabbi et al., 2020a), underscores the critical role of this brain network in the regulation of feeling states/mood states in health and disease. However, there is limited understanding of the molecular biological properties of the AIAC brain network and how specific molecular measures of this brain network might underpin prevalent mood disorder phenotypes and their associated disease burden/morbidity and suicide mortality risk. We, therefore, studied the molecular correlates of mood disorder phenotypes in the AIAC network of postmortem donors using whole tissue RNA-sequencing measures of the association between gene expression changes and disease phenotypes. We studied the gene expression correlates for lifetime mental health, physical health, and mortality outcomes in brain donors with a) a lifetime history of mood disorders and varying psychopathological comorbidity and b) those with no history of psychiatric illness (psychiatrically unaffected controls). We applied a data-driven analysis of the AIAC network's differentially expressed genes (DEGs) to test the hypothesis that this brain network's molecular repertoire will correlate with mood disorders and related comorbid disease phenotypes.

## 2 Methods

**Participants:** This study was approved by the Human Brain Collection Core Oversight Committee. Clinical information on the samples is as follows: Ant-Ins samples included 100 donors, of which 37 BD, 30 MDD, and 33 unaffected controls; and sgACC samples included 152 donors, of which 38 BD, 54 MDD, and 60 unaffected controls. In addition, RNA samples were extracted from the AIAC network's sub-regional *postmortem* tissue using a standardized procedure by the NIMH Human Brain Collection Core (HBCC).

**Brain Dissection, RNA-Extraction, and Sequencing:** The NIMH Human Brain Collection Core (HBCC) provided the *postmortem* samples for which informed consent is acquired according to NIH IRB guidelines. Clinical characterization, neuropathology screening, and toxicology analyses followed previous protocols (Lipska et al., 2006). The region of interest targeted for dissection of the Ant-Ins was defined as the most anterior portion of the insula encompassing the identified reduced gray matter volume (GMV) in the completed meta-analysis by the authors (Jabbi et al., 2020a). Therefore, the dissected regional volume corresponded to the anterior portion of the Ant-Ins, where the caudate and putamen are approximately equal in size (see Supplementary Fig. 1 “Fig. S1A”). Frozen tissue was dissected from the Ant-Ins section for each donor for RNA sequencing. The dissected regional volume from the sgACC was defined as the ACC Brodmann area 32/25 (Fig. S2B) (Akula et al., 2021).

**Illumina-Sequencing, Read-Mapping, and Gene-Quantification of AIAC network:** For the 100 Ant-Ins samples, we processed and sequenced these on the Illumina HiSeq 4000 at the Genome Sequencing and Analysis Facility (GSAF: https://wikis.utexas.edu/display/GSAF/Home+Page) at UT Austin, USA (Supplementary Methods). Thirty million paired-end reads per sample (150 base pairs in length) were generated by sequencing runs of 4 samples per lane of the sequencer. First, sequenced reads were assessed for quality with Fastqc to assess sequencing reads for median base quality, average base quality, sequence duplication, over-represented sequences, and adapter contamination (Andrews, 2010). The reads were pseudo-aligned to the human reference transcriptome (GRCh38-encode) using Kallisto (Bray et al., 2016), and gene-level abundances were obtained.

For the sgACC, the RNA sequencing method and protocol were described earlier in the original study (Akula et al., 2021). We obtained an average of two hundred and seventy million reads per sample, totaling ∼54 billion reads. After quality control, reads were mapped to human genome build 38 using Hisat2 (Pertea et al., 2016). Finally, gene and transcript counts were obtained using StringTie (Pertea et al., 2016). See Supplementary Methods for more details.

### 2.1 Statistical analysis

#### 2.1.1 Factor analysis (our data reduction method) of relevant morbidity and mortality measures

**Postmortem variable factor-analysis:** The postmortem variables included mood disorder diagnoses of MDD or BDD; # of lifetime-Axis-I diagnostic comorbidities or occurrences (e.g., Axis-I-loading of anxiety disorders like generalized anxiety disorder or specific phobias or post-traumatic stress disorders, psychotic and other thought disorders, eating disorders, substance use or poly substance use disorders, etc.); # of lifetime-Axis-III diagnoses (e.g., medical conditions such as diabetes or other metabolic syndromes, cancer, cardiovascular disease, etc.); manner of death (e.g., natural, suicides, homicides or accidents); cause of death as specified by the medical examiner reports (e.g., blunt force trauma to the chest, gunshot, motor vehicle accident, drowning, hanging, etc.); demographics (race, age at death, sex, years of education, number of children/fecundity, and marital records); technical variables (brain-weight, postmortem interval, pH, and RIN-values); and toxicology (blood alcohol/blood narcotics levels). We applied Principal Axis Factoring using the Oblimin Rotation with Kaizer Normalization (Costello and Osborne, 2005) to identify higher-order factors explaining the differences in *postmortem* variables. We included those factors with commonalities of ≥0.45 in follow-up analyses.

Our postmortem metadata variable factor analysis revealed three higher-order factor structures of interest, including *psychiatric morbidity* accounting for 16.27 % of the metadata variance, which is a co-aggregating measure of the following original metadata variables: having a lifetime MDD or BD diagnoses; having an Axis I/psychiatric disorder comorbidity such as psychosis, anxiety disorders, substance/polysubstance use, suicide and increased lethality of the completed suicide; *longevity* accounting for 17 % of the metadata variance, and consisting of a co-aggregating measure of the following original metadata variables: increased Axis-III or medical or physical disease comorbidity, higher age at death despite increased disease load/the presence of medical comorbidity, number of children, and lifetime record of being married versus divorced or single. Since these higher-order factors were generated in a data-driven approach without any priors being determined in terms of which variables should co-aggregate, the identified *longevity* factor is, therefore, not related to suicide measures, which were co-aggregating with *psychiatric morbidity*. Furthermore, the results are from the combined unique donor samples because the results of the separate factor analysis for the Ant-Ins donor samples (72 of which are also in the sgACC samples) and sgACC donor samples were not significantly different. The MDD and BD samples were also not differentiated as our focus was on the composite analysis of higher-order factor-related to molecular changes in comorbid mood disorders (i.e., MDD and BD).

#### 2.1.2 Weighted gene Co-expression Network Analysis (WGCNA), differential gene expression analysis, and AIAC network Rank-Rank Hypergeometric Overlap (RRHO) analysis

Scale-free co-expression networks were constructed with gene abundances using the WGCNA package in R (Langfelder and Horvath, 2008) (See Fig. 1 for data analytics workflow and Figs. 2-4 and Supplementary Figs. 1-7).

**Fig. 1:**
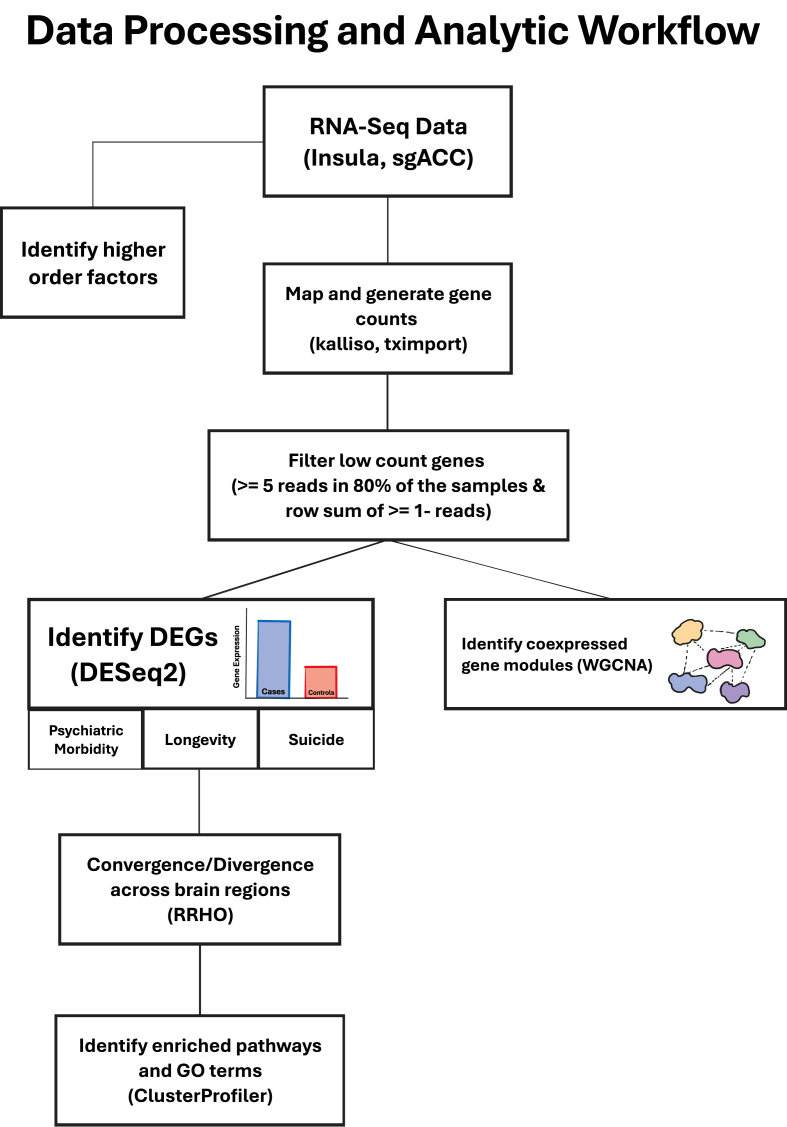
**Analytic Workflow.** From top to bottom, the workflow illustrates the step-by-step processing of RNA-seq data and analysis in the AIAC network donors, including the 180 unique samples.

We then compared gene expression profiles with the two regional datasets by conducting simple comparisons across MDD vs. controls and bipolar disorder vs. controls separately. Differential gene expression between samples differing in the degree of increased psychopathological comorbidity (here referred to as *psychiatric morbidity)* and *longevity* status was assessed across the AIAC network based on the negative binomial distribution for modeled gene counts using DESeq2 (Anders and Huber, 2010). In addition, RNA integrity (RIN) measures or RIN-values were included in our DESeq2 design matrix as a covariate to control for potential confounds. Because we additionally observed a relationship between RIN-values and sex (higher RIN in females than males), this means that any sex effects might be partially covered by the inclusion of RIN in the model as a covariate. As such, we did not correct for sex in the DEG analysis. Only genes with corrected p-value (after Benjamini-Hochberg multiple testing corrections) ≤ 0.05 are reported as significantly differentially expressed. GO-terms enriched in these genes were identified using Enrichr (Kuleshov et al., 2016).

**Fig. 2:**
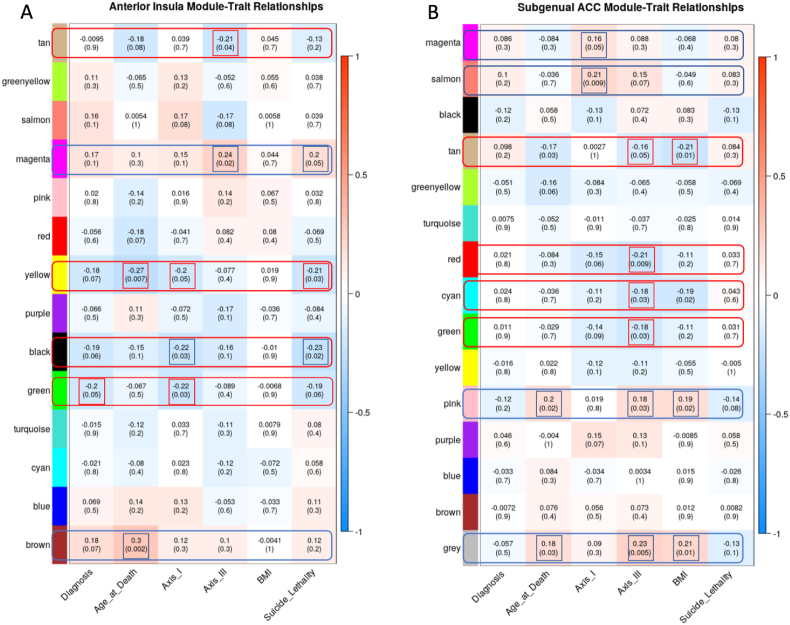
**Weighted Gene Co-expression Network Analysis. A-B**, the y-axis illustrates the WGCNA heatmaps of correlations between specific gene expression modules. The x-axis represents specific clinical phenotypes such as lifetime mental health diagnosis (Diagnosis), age at which the donors died (Age at Death), comorbid lifetime mental disorders (Axis I), comorbid lifetime physical diseases (Axis III), body mass index (BMI), and the lethality of the suicide method for those who completed suicide.

We applied the stratified Rank-Rank Hypergeometric Overlap (RRHO) method implemented by Cahill et al. (2018), an updated and advanced version of previous applications of RRHO using R (Cahill et al., 2018). The updated RRHO algorithm, or “Stratified method,” calculates the degree of overlap based on quadrant-specific analyses (see Fig. 2A–C) (Cahill et al., 2018).

## 3 Results

### 3.1 Demographics, morbidity and mortality variability, and global DEGs

Overall, 100 donors with dissected brain tissue and successful RNA sample extraction from the Ant-Ins region were included in the study: 33 psychiatrically unaffected controls/controls (0 suicide), 37 BD (28 suicide), and 30 MDD (24 suicide) donors. For the sgACC region, 152 samples were included in the study: 60 controls (0 suicide), 38 BD (28 suicide), and 54 MDD (42 suicide) donors. Of the 180 unique donors, 72 were brain donors with both Ant-Ins and sgACC RNA samples extracted and included in the current study (i.e., 72 % of the Ant-Ins samples and 40 % of the sgACC samples consist of both regions).

We found that comorbidity with chronic medical conditions was highest in mood disorders (at F = 5.72, p = 0.004) and more so in MDD vs. controls, followed by bipolar disorder vs. controls, even though a proportion of controls died from terminal Axis-III conditions (Table 1). We further evaluated postmortem body mass index (BMI) differences across all samples and found no association between diagnoses (mood disorders vs. controls) and BMI in the overall Ant-Ins samples. However, the total sgACC samples (including 72 % of the Ant-Ins samples) showed increased BMI in unaffected controls compared with the mood disorder donors (F = 3.7, p = 0.027). See Table 1 for the aggregated BMI statistics in the overall AIAC network samples.

**Tables 1:** Gene expression differences detected at adjusted p < 0.05. Gray-shaded results represent downregulated genes (negative Log2Foldchange values), whereas non-shaded results represent upregulated genes (positive Log2Foldchange values).

Table 1**Psychiatric and Chronic Disease/Medical Comorbidity Presented as % of Comorbid Axis III Diseases**										
**Primary Axis I Diagnosis**	Lung	Cardiovascular	Cancer	Diabetes/Endocrine	Inflammatory/Chronic Pain	Subst. Intoxication/Poisoning	Other CNS	Infection	Obesity (BMI ≥ 25)	# of Donors
Bipolar Disorder (BD)	17.30 %	36.50 %	9.60 %	19.3 %%	15.40 %	42.30 %	19.20 %	5.80 %	71 %	52
Major Depressive Disorder (MDD)	13.30 %	50.00 %	5.00 %	16.7 %%	5.00 %	46.70 %	8.30 %	5.00 %	59.26 %	60
Unaffected controls	8.80 %	74.40 %	7.40 %	13.2 %%	4.40 %	0.00 %	2.90 %	2.90 %	81.66 %	68
Abbreviations: Lung = lung disease; Endocri. = Endocrine diseases/obesity; Subst. = Substance; CNS = Central Nervous System Diseases such as migraine or epilepsy with no focal localization in the AIAC network; # = number						Total # of Unique Donors				180

### 3.2 Weighted gene co-expression network analysis (WGCNA) identifies disease DEG modules

To assess the global gene co-expression profiles for mood disorder diagnoses, other demographics variability, psychiatric disorder and chronic medical disease comorbidity, and suicide mortality-related outcomes across the AIAC network, we performed WGCNA (Langfelder and Horvath, 2008) of the two regions separately. The functionality of the related co-expression pathways was defined using the Gene Ontology (GO) toolbox to identify enriched GO terms (Kuleshov et al., 2016) for each specified WGCNA module.

We further examine gene co-expression beyond the measures of psychiatric phenotypes by assessing Axis III/chronic disease comorbidity-related gene expression modules in the AIAC network (see Fig. 1 for analytic steps). We found that Axis-I and suicide lethality collectively correlated negatively with the yellow module capturing cellular and neuronal ion channel/calcium ion-dependent signaling and synaptic membrane gene co-expression (Schloss and Henn, 2004; Ryding et al., 2006) (Fig. 2A & Fig S3-4), and the black module enriched for O-glycan synthesis and inflammatory cytokine signaling gene co-expression in Ant-Ins (Miller and Raison, 2016). Axis-I psychiatric comorbidity was also correlated positively with the brown module enriched for a wide-ranging inflammatory cytokine response, T-cell immune response, and leukocyte functions gene co-expression in the Ant-Ins (Fig. 2A & Fig S3-4).

We assessed WGCNA for the sgACC data and identified a positive correlation between Axis-I and the salmon module enriched for spliceosome, thyroid hormone, and notch signaling gene co-expression (Fig. 2B & Fig S5-6). On the other hand, Axis-III comorbidity and BMI correlated negatively with the tan module enriched for ribosomal, spliceosomal, mRNA transport and methylation, and protein synthesis (Akula et al., 2021; Pishva et al., 2017) gene co-expression in sgACC (Fig. 2B & Fig S5-6). Furthermore, Axis-III comorbidity correlated negatively with the gray module capturing cellular immune and developmental regulatory gene co-expression in the sgACC (Figs. S5–6). The red, pink, cyan, tan, gray, and green modules known to be enriched for metabolic, protein synthesis, and bodily homeostatic regulatory gene co-expression were also identified in sgACC in association with Axis-III comorbidity and BMI (Fig. 2B & Figs. S5–6).

### 3.3 Mood disorder-specific differentially gene expression analysis identified DEGs

Using a statistical threshold of q = 0.05 adjusted for multiple comparisons using false discovery rate correction (FDR) (Benjamini and Hochberg, 1995), we assessed differential gene expression in MDD versus (vs.) controls and in BD vs. controls to identify diagnosis-specific DEGs across the Ant-Ins and the sgACC regions. Our identified DEGs in the Ant-Ins in MDD vs. controls included five genes, namely: a downregulated *SELE* gene known to control leukocyte regulation of inflammation, and four upregulated genes including the cytokine interleukin-1 receptor-like *IL1RL1* gene, a gene that regulates *IL-33/ST2* (Uversky, 2014), a phosphorylated protein binding *FBXO47* gene, a mitochondrial electron transporter *MTCO2P12*, and a long-noncoding RNA (lncRNA) H19 in the Ant-Ins (Supplementary-Table-1A (TableS1A)). To compare BD vs. controls, we found one lncRNA RP1-193H18.3 to be upregulated (Table S1B). Furthermore, we found no DEGs for MDD vs. controls or BD vs. controls in the sgACC at the adjusted p-value of 0.05 FDR.

### 3.4 Factor analysis (the adopted data reduction method) identified relevant morbidity and mortality indicators

To better examine the inter-relationship between complex disease comorbidity and underlying brain molecular pathology as measured in the AIAC network gene expression using whole tissue RNA-seq in donors who died of both chronic medical conditions (Axis-III) and mood disorders related to suicide, we applied a factor analytic data reduction to identify hidden phenotypic variability in our data that may influence DEGs. The application of a factor analysis of the postmortem phenotypic data is crucial because it allows a novel data-driven method of assessing what aggregate/composite variabilities could be driving biological gene expression changes (DEGs) in the studied samples without relying on predefined variables like diagnosis, age or sex which may not be sufficiently driving biological variability related to mood disorder metrics. To this aim, we included diagnoses, Axis-I, Axis-III, BMI, age at death, and suicide lethality variables, etc., in a factor analytical model using principal axis factoring for identifying higher-order variables that are more sensitive for precise quantification of phenotype-related DEGs (Jabbi et al., 2020a; Arasappan et al., 2021). See Supplementary Methods and Results for details of how factor analysis results guided RNA-seq analytics.

### 3.5 Psychiatric (co)morbidity-related differential gene expression analysis identified DEGs

We first assessed DEGs associated with *psychiatric morbidity*. All humans, including healthy people, often undergo periodic experiences of positive and negative mood changes throughout their lifespans. We included the controls in our factor analysis and initial differential gene expression analyses that assessed transcript abundance associated with *psychiatric morbidity* and related phenotypes independent of diagnoses. Controls were further removed from secondary analysis to evaluate DEGs associated with *psychiatric morbidity* within the mood disorder samples.

**Fig. 3:**
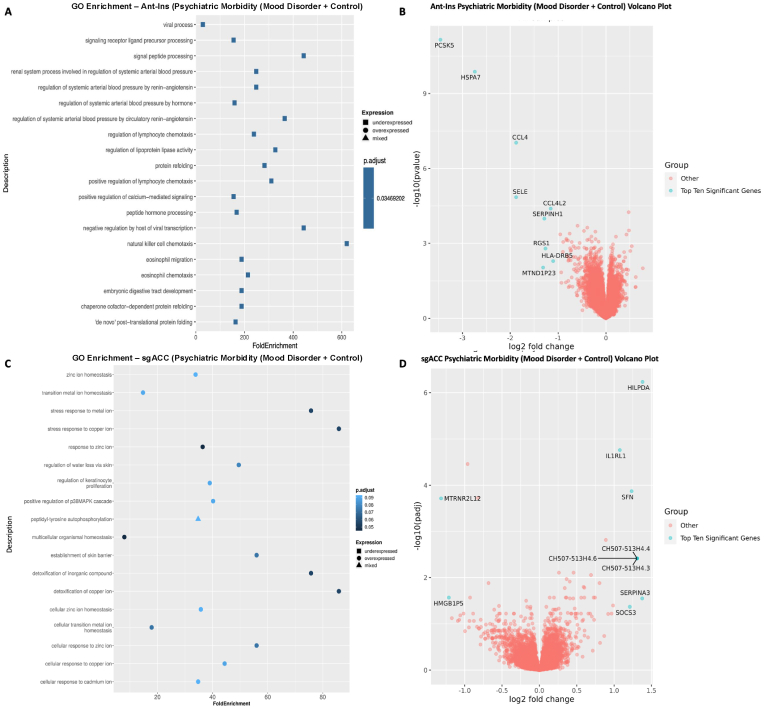
**Gene Ontology (GO) terms and Volcano plots for High vs. Low Psychiatric Morbidity (higher-order factor loadings were used to compare high comorbid/morbidity versus low morbidity individuals in a differential gene expression analysis)**. **A**) illustrates GO terms for GECs in Ant-Ins for high vs. low psychiatric morbidity, with **B**) depicting the related volcano plot for the Ant-Ins results in **A**. **C & D**) illustrates GO terms in sgACC representing high vs. low psychiatric morbidity and related volcano plots for the sgACC results in **C**. Because the -log10 (adjusted q-values) of a large number of genes in Insula was close to zero, volcano plots were generated using q-value instead of adjusted q-value. Genes meeting the following cutoffs, adjusted q-value <0.05, and absolute log2 fold change ≥ 1 were highlighted on the volcano plot as significant genes.

Using this approach, we then applied a median split-half method of identifying DEGs associated with high vs. low psychiatric morbidity (including MDD, BD, and unaffected control samples in our analytic model) across the AIAC network at p ≤ 0.05 FDR. In the Ant-Ins, we found three downregulated DEGs that recapitulated our mood disorder vs. control findings (see Table S2), including the protein synthesis *PSK5* gene and ATP-binding heat shock protein *HSPA7* gene (Jabbi et al., 2020a; Pantazatos et al., 2017) and a mitogen-inducible monokine called C-C motif chemokine ligand-4 immunoregulatory and inflammatory *CCL4* gene (Table S2A; Fig. 3A and B). We then performed a secondary analysis comparing high vs. low *psychiatric morbidity* in the mood disorder samples (excluding controls) to assess if our identified Ant-Ins DEGs are proximate to mood pathology. For this mood disorder-specific analysis, we found two of the three downregulated genes in the mood disorders and control analysis, including *PSK5* and *HSPA7* and the mitochondrial electron transporter *MTCO2P12*, surviving p ≤ 0.01 FDR (Table S2A; Fig. 3A and B). Further, our observed high *psychiatric morbidity* GO-terms were enriched for immune, protein synthesis, complement activation, and Fc-gamma receptor signaling DEGs in Ant-Ins (Laguesse and Ron, 2020) (Fig. 3A and B).

Differential gene expression analysis of sgACC samples for high vs. low *psychiatric morbidity* in all samples, including mood disorders and controls, yielded forty-seven DEGs, including twelve downregulated DEGs (Table S2B; Fig. 3C and D). DEGs for high vs. low *psychiatric morbidity* in sgACC also revealed thirty-five upregulated genes, including the autocrine signaling lipid storage and metabolism gene *HILPDA* implicated in stress responsiveness/physical activity/energy expenditure (Cantarelli et al., 2014; VandeKopple et al., 2017), interleukin 1 receptor-like *IL1RL1* (Uversky, 2014), mitotic translational regulator *SFN*, synaptic membrane/calcium ion channel *MT1X* (Sokolowski et al., 2018; Zandi et al., 2022), an uncharacterized protein *C3orf20*, Calcium-dependent adhesion protein *CDH3* genes, the iron homeostatic hepcidin antimicrobial peptide *HAMP* gene, and the CH507-513H4.3, CH507-513H4.4, and CH507-513H4.6 novel transcripts, etc. (Table S2B; Fig. 3C and D). Of interest, replicating the high vs. low *psychiatric morbidity* comparison in the mood disorder samples only (excluding controls) yielded no DEGs in the sgACC, as if the sgACC gene regulatory repertoire likely underpins the presence of mood disorder diagnosis rather than the graded disease morbidity or severity. On the other hand, the GO-terms for *psychiatric morbidity-associated* DEGs in all samples of the sgACC identified enriched pathways for cellular signaling, zinc ion homeostasis, multicellular organismal homeostasis, and metabolic balance (Zhang et al., 2022) (Fig. 3C and D). Notably, although the unaffected controls have no recorded mental disorder history, including them in the high vs. low *psychiatric morbidity* analysis did not dampen the number of DEGs in the studied network.

### 3.6 Suicide completion-related differential gene expression analysis identified DEGs

**Fig. 4:**
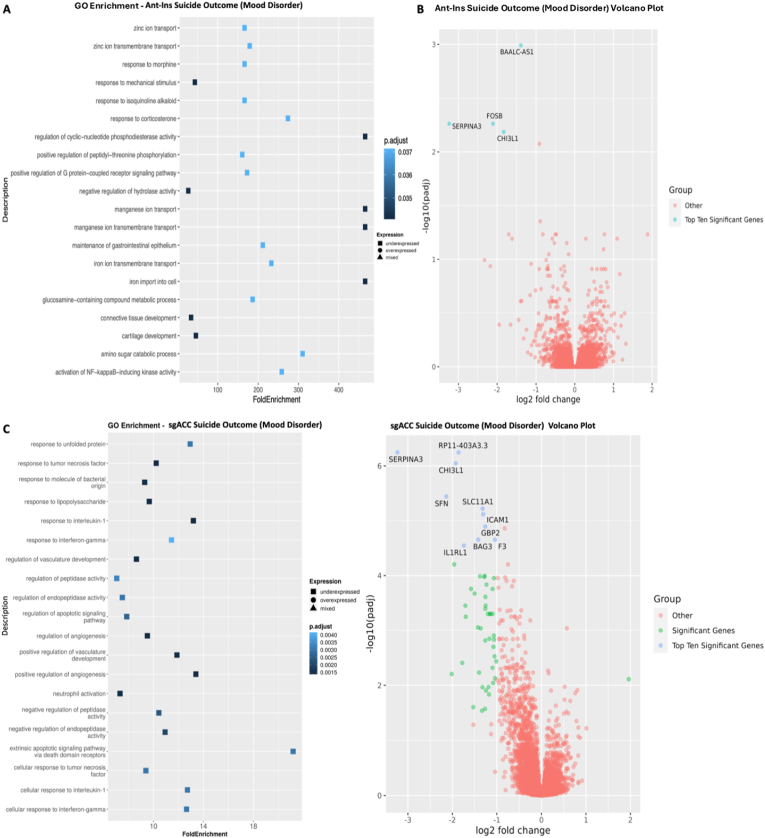
**Gene Ontology (GO) terms and Volcano plots for Suicide Completions vs. Non-Suicide Deaths**. **A**) illustrates GO terms for GECs in Ant-Ins for suicide completion vs. non-suicide deaths, with **B**) depicting the related volcano plot for the Ant-Ins results in **A**. **C & D**) illustrates GO terms in sgACC representing suicide completion vs. non-suicide deaths and related volcano plots for the sgACC results in **C**. Because the -log10 (adjusted q-values) of a large number of genes in Insula was close to zero, volcano plots were generated using q-value instead of adjusted q-value. Genes meeting the following cutoffs, adjusted q-value <0.05, and absolute log2 fold change ≥ 1 were highlighted on the volcano plot as significant genes.

Because a significant percentage of our studied mood disorder samples died by suicide/are suicide completers (∼60+% of the included mood disorder donor samples), this makes suicide a significant predictor of premature death and underlying DEGs in our disease samples. We, therefore, quantified DEGs for suicide completion vs. non-suicide deaths (excluding controls which, by default, had no mental disorder or suicide history) at p ≤ 0.05 FDR. We found six downregulated Ant-Ins DEGs, including the cell growth inhibiting serpine family *SERPINA3* gene that was earlier implicated in schizophrenia, *FOSB* transcription factor involved in encoding leucine zipper proteins and dimerization of proteins of the JUN family, thereby regulating leukocyte and T-cell proliferation, differentiation, and transformation (Heximer et al., 1996; Baumann et al., 2003), inflammation and tissue remodeling *CHI3L1* gene found to be associated with Alzheimer's disease and schizophrenia (Ohi et al., 2010), and a BAALC-AS1 lncRNA (Punzi et al., 2019) (Table S3A, Fig. 4A and B), and a long non-coding RNA AC145676.2, etc. The GO-terms for Ant-Ins DEGs in suicides were enriched for tyrosine-protein processing, B-cell activation, immune response, and regulatory T-cell pathways (Fig. 4A and B).

Our analysis of suicide-completed associated DEGs in the sgACC (excluding controls) identified predominantly downregulated markers (i.e., 313 of the total 332 markers were downregulated), including the cell growth inhibiting serpine family of proteins *SERPINA3, SERPINE1*, and *SERPINA1*, and the inflammation mediator *CHI3L1* (also downregulated in Ant-Ins in suicide completers). Furthermore, the interleukin *IL1RL1* and *HILPDA* autocrine signaling lipid storage genes (Cantarelli et al., 2014) were also downregulated, alongside an additional 307 downregulated DEGs in the sgACC of suicide completers. Conversely, the N-acetyltransferase meta-pathway biotransformation I and II gene, *NAA40*, the helix-loop transcriptional regulator *NPAS4*, and 17 other DEGs were selectively upregulated in sgACC of suicide death cases (Table S3B; Fig. 4C and D). GO-terms for suicide completion-associated DEGs in sgACC were enriched for innate and adaptive immune/inflammatory, cell-type mediated tissue remodeling, and apoptosis regulatory genes (Raja et al., 2022) (Fig. 4C and D). For results of overlaps between the Ant-Ins and sgACC, see our Rank-Rank Hypergeometric findings (*RRHO,* Fig. 2A–C) which identified significant gene expression overlaps within the AIAC network (Joyce and Barbas, 2018; Wang et al., 2021), especially the extratelencephalic cellular links between the AIAC network and cardinal peripheral organs like the heart, gut, and adrenal gland (Dum et al., 2019; Levinthal and Strick, 2020).

***Ant-Ins and sgACC Rank-Rank Hypergeometric Overlap (RRHO) in Psychiatric Morbidity, Suicide Completion, and Longevity:*** In line with previous neuroimaging findings of similar patterns of reduced anatomical gray matter reductions in neuropsychiatric diagnoses, suicidal phenotypes, and aging, we found a preponderance of gene expression overlap across the AIAC network. Specifically, we found both downregulated and upregulated gene expression overlaps across the AIAC network such that all three contrasts of interest showed extensive interregional overlap. However, the degree of overlapping downregulated gene expression was highest in suicide completion and longevity-associated DEGs, respectively (Fig. 2A–C).

## 4 Discussion

This study identified patterns of differentially expressed genes (DEGs) underlying mood disorders and co-occurring psychiatric disorders. We demonstrated that it is feasible to identify specific DEGs for complex disease phenotypes like *psychiatric morbidity* and suicide outcomes (which are composite measures of maladaptive mood and related behavioral outcomes) in addition to *longevity*. We applied postmortem brain RNA-sequencing of the AIAC brain network, known to regulate mood states in health and disease (Nauta, 1972; Goldman-Rakic, 1988; Drevets et al., 2008) and harbors reduced anatomical integrity in mood and comorbid psychiatric disorders (Drevets et al., 2008; Goodkind et al., 2015; Wise et al., 2017; Jabbi et al., 2020a) and suicidal phenotypes (Schmaal et al., 2020; Jabbi et al., 2020b). The targeted AIAC network exhibits anatomical and physiological changes associated with therapeutic responses (McGrath et al., 2013; Riva-Posse et al., 2018), and transcriptome studies of this brain network revealed molecular abnormalities related to mood disorders and suicide phenotypes (Jabbi et al., 2020a; Galvalvy et al., 2013; Orozco-Solis et al., 2017; Lutz et al., 2017; Zhou et al., 2018).

Our WGCNA results reveal significant negative correlations between *psychiatric morbidity* and related suicide variables in terms of critical gene expression modules related to synaptic membrane and ion channel signaling (Akula et al., 2021; Pishva et al., 2017; Sokolowski et al., 2018; Zandi et al., 2022); homeostatic regulatory processes like inflammatory signaling (Arasappan et al., 2021; Elhaik and Zandi, 2015; Schiweck et al., 2020); and metabolic processes like mitochondrial translation (Laguesse and Ron, 2020); and ATP-synthesis pathways (Jabbi et al., 2020a; Pantazatos et al., 2017). Our observed WGCNA in the sgACC showed correlations between *psychiatric morbidity* and *longevity* with molecular processes like enriched protein synthesis (Akula et al., 2021; Pishva et al., 2017), basic cellular processes, and neurodegeneration (Raja et al., 2022; Glover et al., 2018). Together, these results suggest an AIAC network regulation of a broad biological process encompassing cellular, metabolic, and immune mechanisms critical for engendering adaptive behaviors, including mood phenotypes. Likely, our observed negative correlation between the AIAC network WGCNA captured gene expression modules signifies a potential collective decline in the functionality of the AIAC network, at least at the molecular level, in *psychiatric* and suicide phenotypes.

Although few postmortem brain studies (Turecki et al., 2019), including our recent work (Jabbi et al., 2020a; Akula et al., 2021; Arasappan et al., 2021) have characterized maladaptive (lifetime morbidity and suicide mortality), relative to adaptive (longevity despite lifetime psychiatric and other diseases) phenotypes in brain transcriptomes of mood disorders, our current direct comparisons of MDD vs. controls and BD vs. controls identified fewer mood disorder-specific DEGs relative to our comparisons of higher-order factors like high vs. low *psychiatric morbidity* and high vs. low *longevity* across the AIAC network. Notably, excluding controls in our analysis revealed preserved downregulated protein synthesis DEGs, suggesting a more perversive protein synthesis dysregulation in increased *psychiatric morbidity* than previously thought. Further analysis of high vs. low *psychiatric morbidity* in the sgACC yielded downregulated G-protein-coupled DEGs and a predominant upregulation of metabolic, stress-responsive (Cantarelli et al., 2014; VandeKopple et al., 2017), inflammatory, and synaptic membrane/calcium ion channel (Sokolowski et al., 2018), and iron homeostasis DEGs.

By assessing DEGs for suicide completion, we uncovered predominantly downregulated Ant-Ins DEGs associated with protein synthesis, immune and inflammatory signaling, B-cell activation (Miller and Raison, 2016), and synaptic membrane regulatory functions (Sokolowski et al., 2018; Zandi et al., 2022). Similarly, sgACC DEGs associated with suicide completion were predominantly downregulated innate and adaptive immune pathway functions (Uversky, 2014; Mechawar et al., 2016; Giridharan et al., 2020), tissue/cellular development and apoptosis regulatory pathways, and translational regulatory pathways. Given that increased *psychiatric morbidity* is the most predominant risk factor for suicide, these findings can be interpreted in two ways. *First*, Adverse life experiences and trauma/stress are likely causally linked to mood disorders and related downregulations in AIAC network gene expression, and with increased brain synaptic imbalances that can trigger microglial mediated overrunning of stress-damaged neurons or neuronal debris and result in loss of neuronal connectivity and synaptic membrane gene regulatory functions, with potential developmental and lifelong impaired functional consequences (Raja et al., 2022; Glover et al., 2018). *Second*, our findings of neurodevelopmental, tissue developmental, and synaptic/ion channel regulatory DEGs can be meaningful both in terms of early developmental gene-mediated neurodevelopmental deficits leading to lifelong repercussions like increased *psychiatric morbidity* and suicide risk outcomes (Turecki et al., 2019; Jabbi et al., 2020a). Most importantly, cellular and neurodevelopmental gene regulatory abnormalities may comprise a neurobiological vulnerability that manifests as a mood disorder when individuals with these developmental gene-regulation deficits are further exposed to early life adversities, as is often the case for individuals with high familial/genetic risk for mood disorders (Cantarelli et al., 2014; Slavich et al., 2010; Cox et al., 2021).

Here, we tested the hypothesis that the phenotypic complexity of comorbid psychiatric diseases in individuals with primary mood disorders may confer immune and inflammatory-related neuropathological markers that may also be involved in longevity/aging and related lifetime chronic physical disease. Given that mood disorders are triggered by traumatic experiences which can cause a long-term cascade of inflammation and associated physical harm, it is likely that the overlap in the downregulated immune and inflammatory responses related to both mental and physical diseases identified in our samples underscores shared pathobiological processes related to immune clearance of disease induced homeostatic imbalances/inflammation emanating from both experienced trauma and physical diseases. Furthermore, our findings of downregulated protein synthesis and ion/calcium channel signaling DEGs in high vs. low *psychiatric morbidity*, shown to be spread across the AIAC network, support our hypothesis and could serve as a possible underlying mechanism for the often-observed network anatomical integrity reductions in mood disorders and comorbid conditions (Goodkind et al., 2015; Wise et al., 2017; Jabbi et al., 2020a).

The current study has limitations in that even though we downsampled the sgACC data to be comparable with the Ant-Ins data, the RNA-seq methods were not identical across the two regions, so we cannot preclude the possibility that some differences we observed between Ant-ins and sgACC regions are driven by methodologic differences. However, such differences cannot explain the convergent signals we observed. Secondly, although 72 of the 152 donors we studied contributed data from both Ant-Ins and sgACC regions, there were more donors of sgACC tissue. This imbalance means that the sources of variability likely differed across the two brain regions, reducing comparability. Thirdly, bulk RNA sequencing cannot account for cell-type-specific differences in gene expression signatures in terms of which cell types drive specific downregulation or upregulation of key transcriptional elements. Future studies that apply novel single-cell approaches using the higher-order variability analytic approaches defined in our research will be needed to identify dosage-sensitive cell-type specific neuropathological influences at transcriptomic scales and guide novel diagnostic and therapeutic advances. These future cell-type studies will be critical as the Ant-Ins and sgACC share several cell population phenotypes (Dum et al., 2019; Joyce and Barbas, 2018). Finally, although we included RIN values as covariates, we did not correct for sex because it co-aggregated with RIN values with female donors found to have higher RIN values than male donors in our studied cohort. Such a limitation of not covarying for sex warrants future examination of sex effects that may be related to or even be causally link to immune, inflammatory, and other sex-related gene regulatory signaling pathways.

Our findings of an association between chronic *psychiatric morbidity* (higher numbers of lifetime *psychiatric disease comorbidity*, and completed suicide, with a preponderance of downregulated protein synthesis and inflammatory, cellular developmental, and metabolic DEGs, likely underscores the vital role of these molecular mechanisms in the maintenance of brain and body homeostasis in health and diseases. The morbidity and mortality-related gene expression changes highlight key immune-metabolic and cellular signaling pathways within a critical AIAC brain network involved in emotional and mood regulatory functions. In conclusion, our findings provide a mechanistic framework for understanding dosage-dependent (i.e., downregulated vs. upregulated) gene expression repertoires for adaptive and maladaptive mood functions. The results could inform novel diagnostic and therapeutic innovations for comorbid psychiatric disease phenotypes and suicide mortality outcomes across the lifespan.

## CRediT authorship contribution statement

**Dhivya Arasappan:** Writing – review & editing, Resources, Investigation, Data curation, Software, Methodology, Formal analysis, Conceptualization. **Abigail Spears:** Methodology, Writing – review & editing, Formal analysis. **Simran Shah:** Writing – review & editing, Formal analysis, Methodology. **Roy D. Mayfield:** Writing – review & editing, Formal analysis, Methodology, Data curation. **Nirmala Akula:** Methodology, Funding acquisition, Data curation, Writing – review & editing, Investigation, Formal analysis, Conceptualization. **Francis J. McMahon:** Writing – review & editing, Methodology, Funding acquisition, Conceptualization, Resources, Investigation, Data curation. **Mbemba Jabbi:** Writing – original draft, Validation, Resources, Methodology, Funding acquisition, Data curation, Visualization, Supervision, Project administration, Investigation, Formal analysis, Conceptualization.

## Declaration of competing interest

The Above listed Authors hereby declared their joint interest of pursuing the peer-reviewed publication of the manuscript titles: Brain transcriptomic signatures for mood disorders and suicide phenotypes: an anterior insula and subgenual ACC network postmortem study.

Dr. Mbemba Jabbi, is signing this declaration of interest on behalf of the authorship team.

## Data Availability

Data will be made available on request.
