# Supplementary material for: Brain transcriptomic signatures for mood disorders and suicide phenotypes: an anterior insula and subgenual ACC network postmortem study
Source: Brain Behav Immun Health. 2025 Jul 5;48:101051. doi: 10.1016/j.bbih.2025.101051 (PMC12274802; doi:10.1016/j.bbih.2025.101051)
Supplement: Multimedia component 1 [file mmc1.docx]

**Brain transcriptomic signatures for mood disorders and suicide phenotypes: an anterior insula and subgenual ACC network postmortem study**

**SUPPLEMENTARY METHODS**

**RNA-Extraction of Ant-Ins & sgACC:** The HBCC further pulverized all dissected tissues separately and aliquoted 50mg from each sample for standardized total RNA processing. Specifically, RNeasy Lipid Tissue Mini Kit (50) was used for RNA purification using the 50 RNeasy Mini Spin Columns, Collection Tubes (1.5 ml and 2 ml), QIAzol Lysis Reagent, RNase-free Reagents, and Buffers kit from Qiagen. DNase treatment was applied to the purified RNA using a Qiagen RNase-Free DNase Set (50) kit consisting of 1500 Kunitz units RNase-free DNase I, RNase-free Buffer RDD, and RNase-free water for 50 RNA minipreps. After DNAse treatment, the purified RNA from pulverized AIAC was used separately per individual to determine RNA quality as measured in RNA integrity number (RIN) values using Agilent 6000 RNA Nano Kit consisting of the microfluidic chips, Agilent 6000 RNA Nano ladder, and reagents on Agilent 2100 Bioanalyzer. Samples with RIN < 6 were excluded from the study.

**Illumina-Sequencing, Read-Mapping, and Gene-Quantification of AIAC network:** For the 100 Ant-Ins samples, we processed and sequenced these on the Illumina HiSeq 4000 at the Genome Sequencing and Analysis Facility (GSAF: <https://wikis.utexas.edu/display/GSAF/Home+Page>) at UT Austin, USA (Supplementary Methods). Thirty million paired-end reads per sample (150 base pairs in length) were generated by sequencing runs of 4 samples per lane of the sequencer. First, sequenced reads were assessed for quality with Fastqc to assess sequencing reads for median base quality, average base quality, sequence duplication, over-represented sequences, and adapter contamination [25]. We looked at median base quality, average base quality, sequence duplication, over-represented sequences, and adapter contamination, which were < 5%. Median quality at every base was > 30 for all samples, and more than 90% of the reads had average base quality > 30, making read trimming or filtering redundant. We did not remove samples because all samples had typical sequence duplications < 60%, as in high-coverage RNA-Seq data, and no adaptor trimming was performed (i.e., adaptor contamination percentages < 5%). Next, the reads were pseudo-aligned to the human reference transcriptome (GRCh38-encode) using Kallisto [26], and gene-level abundances were obtained.

For the sgACC, the RNA sequencing method and protocol were described earlier in the original study [24]. Briefly, total RNA extracted from frozen dissections of sgACC and only samples with RNA integrity numbers (RIN values) greater than 6, as confirmed using the Agilent Bioanalyzer, were used for library preparation. Total RNA was captured using the RiboZero protocol, followed by library preparation, and stranded paired-end sequencing was performed on the RNA samples using the Illumina HiSeq 2500 system. We obtained an average of two hundred and seventy million reads per sample, totaling ~54 billion reads. After quality control, reads were mapped to human genome build 38 using Hisat2 [27]. Finally, gene and transcript counts were obtained using StringTie [27]. For both Ant-Ins and sgACC, any genes expressing 0 in 80% or more samples were filtered out to remove low-count genes from further analysis. Next, the abundances were normalized using DESeq2 and transformed with variance stabilizing transformation (a transformation to yield counts that are approximately homoscedastic, having a constant variance regardless of the mean expression value). Finally, Principal Component Analysis was performed using 25% of the highest variance genes to explore the underlying data’s structure and the most significant sources of variance. Lastly, genes with an expression value of 0 in 80% of samples or more were removed from further analysis to correct for sporadically large fold-change outliers. See Supplementary Methods for more details.

**Weighted Gene Co-Expression Network Analysis (WGCNA):** Scale-free co-expression networks were constructed with gene abundances using the WGCNA package in R [28] (See **Fig 1** for data analytics workflow). WGCNA provides a global perspective and allows the identification of co-expressed gene modules. It avoids relying on arbitrary cutoffs involved in selecting differentially expressed genes. Instead, it identifies a group of genes changing in the same direction and magnitude, even if these changes are smaller. WGCNA identifies co-expressed modules of genes, thereby identifying genes that are likely co-regulated or may belong to the same functional pathway, using a dynamic tree-cutting algorithm based on hierarchical clustering (i.e., minimum module size=30). A given module’s eigengene, defined as the first principal component of the expression matrix of the corresponding module, can be correlated to sample variables to identify modules of interest. We correlated the module eigengenes to different postmortem sample characteristics and selected the two modules that showed significant correlation to variables of interest, such as *diagnostic and suicide-linked variables*. Driver genes (i.e., genes within co-expressed gene modules whose distinct expression patterns are similar to the overall expression profile of the entire co-expressed modules) were used to identify pathobiological functions associated with each module.

**Differential Gene Expression Analysis:** We first compared gene expression profiles with the two regional datasets by comparing MDD vs. controls and bipolar disorder vs. controls separately.

Our exploratory factor analysis assessed the relationship between all the postmortem variables to determine the existence of higher-order factor loadings that better explain postmortem variance than the original variables (see results section for full details). Two filters were used to remove low signal or outlier genes from the differential expression analysis. Besides the bipolar > controls and MDD > controls contrasts, only genes with 5 reads or more in 80% of the samples and a row sum of 10 reads or more were considered for further analysis. These filters help prevent undetected genes in most samples and lowly expressed in a few samples from being identified as differentially expressed genes. To identify gene expression signatures related to differences in our identified higher-order factors, such as psychiatric morbidity, we compared high (samples scoring above the median split of the factor loading) scores on psychiatric morbidity vs. low (samples scoring below the median split of the factor loadings) scores of this factor. Similar comparisons were carried out for longevity. Differential gene expression between samples differing in psychiatric morbidity and longevity status was assessed across the AIAC network based on the negative binomial distribution for modeled gene counts using DESeq2 [30]. In addition, RIN-values were included in the DESeq2 design matrix as a covariate to control for potential confounds.

Controls were omitted in the last comparison (i.e., to examine gene expression profiles that might be linked explicitly to suicide completion vs. non-suicide deaths in persons diagnosed with mood and comorbid psychiatric disorders. Therefore, only genes with corrected p-value (after Benjamini-Hochberg multiple testing corrections) ≤ 0.05 are reported as significantly differentially expressed. GO-terms enriched in these genes were identified using Enrichr [31].

**AIAC network Rank Rank Hypergeometric Overlap (RRHO) analysis:** We applied the stratified RRHO method implemented by Cahill et al. [32], an updated and advanced version of previous applications of RRHO using R [32]. In essence, the updated RRHO algorithm is designed to quantify the preponderance (significance) of correlation or overlap between two gene lists from two sets of independent experiments or datasets like our examples of the datasets from the AIAC network in the current study in terms of upregulation or downregulation based on enrichment measures. The updated RRHO algorithm, or “Stratified method,” calculates the degree of overlap based on quadrant-specific analyses (see **Fig 5A-C**) [32]. Precisely, the updated method designed a new approach that takes each quadrant and counts from the outward corner to the cutoff point to define the number of genes from the first gene expression dataset (Ant-Ins) and the number of genes from the second dataset (sgACC) and assess the overlapping enrichment between them.

**SUPPLEMENTARY RESULTS**

**Demographics, morbidity and mortality variability, and global DEGs.**

Overall, 100 donors with dissected brain tissue and successful RNA sample extraction from the Ant-Ins region were included in the study: 33 psychiatrically unaffected controls/controls (0 suicide), 37 BD (28 suicide), and 30 MDD (24 suicide) donors. For the sgACC region, 152 samples were included in the study: 60 controls (0 suicide), 38 BD (28 suicide), and 54 MDD (42 suicide) donors. Of the 180 unique donors, 72 were brain donors with both Ant-Ins and sgACC RNA samples extracted and included in the current study (i.e., 72% of the Ant-Ins samples and 40% of the sgACC samples include both regions).

To first examine the degree of mood disorder co-occurrence with other psychiatric (Axis-I) and medical (Axis-III) conditions, we used an analysis of variance (ANOVA) to assess if the presence of chronic medical conditions like cardiovascular diseases, cancers, and diabetes differ between BD, MDD, and controls. We found that comorbidity with chronic medical conditions was highest in mood disorders (at F=5.72, p=0.004) and more so in MDD vs. controls, followed by bipolar disorder vs. controls, even though a proportion of controls died from terminal Axis-III conditions (**Table 1**). We then assessed the degree of Axis I comorbidities like having lifetime BD with co-occurring anxiety, polysubstance use, psychosis in the same donor or having lifetime MDD with co-occurring psychosis, anxiety, post-traumatic stress disorder, alcohol use disorder all in the same donor (degree of psychiatric comorbidity across the samples). We found no differences between the MDD and BD samples when comparing the Ant-Ins and sgACC regional datasets. We further evaluated postmortem body mass index (BMI) differences across all samples and found no association between diagnoses (mood disorders vs. controls) and BMI in the overall Ant-Ins samples. However, the total sgACC samples (including 72% of the Ant-Ins samples) showed increased BMI in unaffected controls compared with the mood disorder donors (F=3.7, p=0.027).

**Factor analysis (our data reduction method) of relevant morbidity and mortality measures.**

To better examine the inter-relationship between complex disease comorbidity and underlying brain molecular pathology as measured in the AIAC network gene expression using whole tissue RNA-seq in donors who died of both chronic medical conditions (Axis-III), mood disorders related to suicide, we applied a factor analytic data reduction to identify hidden phenotypic variability in our data that may influence DEGs. The application of a factor analysis of the postmortem phenotypic data is crucial because it allows a data-driven method of assessing what aggregate/composite variabilities could be driving biological gene expression changes (DEGs) in the studied sample without relying on predefined variables like diagnosis, age or sex which may not be sufficiently driving biological variability related to mood disorder metrics. To this aim, we included diagnoses, Axis-I, Axis-III, BMI, age at death, and suicide lethality variables, etc., in a factor analytical model using principal axis factoring for identifying higher-order variables that are more sensitive for precise quantification of phenotype-related DEGs [22,39]. See Supplementary Results for details of how factor analysis results guided RNA-seq analytics.

**Weighted gene co-expression network analysis (WGCNA) identifies disease DEG modules.**

To assess the global gene co-expression profiles for mood disorder diagnoses, other demographics variability, psychiatric disorder and chronic medical disease comorbidity, and suicide mortality-related outcomes across the AIAC network, we performed WGCNA [28] of the two regions separately. The functionality of the related co-expression pathways was defined using the Gene Ontology (GO) toolbox to identify enriched GO terms [31] for each specified WGCNA module.

We further examine gene co-expression beyond the measures of psychiatric phenotypes by assessing Axis II/chronic disease comorbidity-related gene expression modules in the AIAC network (see **Fig 1** for analytic steps). We found that Axis-I and suicide lethality collectively correlated negatively with the yellow module capturing cellular and neuronal ion channel/calcium ion-dependent signaling and synaptic membrane gene co-expression [33,34] (**Fig 2A & Fig S3-4**), and the black module enriched for O-glycan synthesis and inflammatory cytokine signaling gene co-expression in Ant-Ins [35]. Axis-I psychiatric comorbidity was also correlated positively with the brown module enriched for a wide-ranging inflammatory cytokine response, T-cell immune response, and leukocyte functions gene co-expression in the Ant-Ins (**Fig 2A & Fig S3-4**).

We assessed WGCNA for the sgACC data and identified a positive correlation between Axis-I and the salmon module enriched for spliceosome, thyroid hormone, and notch signaling gene co-expression (**Fig 2B & Fig S5-6)**. On the other hand, Axis-III comorbidity and BMI correlated negatively with the tan module enriched for ribosomal, spliceosomal, mRNA transport and methylation, and protein synthesis [24,36] gene co-expression in sgACC (**Fig 2B & Fig S5-6)**. Furthermore, Axis-III comorbidity correlated negatively with the grey module capturing cellular immune and developmental regulatory gene co-expression in the sgACC (**Fig S5-6**). The red, pink, cyan, tan, grey, and green modules known to be enriched for metabolic, protein synthesis, and bodily homeostatic regulatory gene co-expression were also identified in sgACC in association with Axis-III comorbidity and BMI (**Fig 2B & Fig S5-6**).

**Differential Gene Expression Validation.**

Given that our median split-half method included all samples, we conducted an additional Ant-Ins analysis of DEGs by comparing the subgroup of donors at the two extremes (i.e., 20 donors with the lowest scores on *psychiatric morbidity* vs. 20 donors with the highest scores on *psychiatric morbidity*). First, we compared *psychiatric morbidity* for the 20 samples with the lowest psychiatric morbidity vs. 20 with the highest psychiatric morbidity using adjusted p<0.05 FDR cutoff FDR. As a result, we found one downregulated DEG, namely the Neuronal PAS Domain Protein 4 master transcriptional regulator (NPAS4), involved in various biological functions, including physiological and developmental events gene (**Table S6A**).

We then repeated a similar analysis comparing the 20 lowest psychiatric morbidity samples in the mood disorder cohort (excluding controls) vs. the 20 highest psychiatric morbidities in the mood disorder samples. We found no DEGs surviving p<=0.05 FDR, suggesting that Ant-Ins DEGs may not be sensitive to extreme differences in measures of *psychiatric morbidity within disease samples* (**Table S6B**). Our additional comparison of *psychiatric morbidity* scores in the lowest and highest extremes, in the totality of all samples, and comparing the 20 lowest vs. the 20 highest psychiatric morbidity scores in mood disorder samples alone resulted in more DEGs in the sgACC, unlike the Ant-Ins that did not show any highest 20 vs. lowest 20 scoring *psychiatric morbidity* related DEGs. These findings suggest that such extreme comparisons may reveal related and unique molecular profiles likely differentially mediated in each brain network node.

At the global level, we found more differentially expressed genes (DEGs) in the sgACC region compared to Ant-Ins when using psychiatric comorbidity when comparing gene expression in suicide completer vs. non-suicide deaths as contrasts of interest. Using psychiatric comorbidity (one of two data reduction identified factors, see methods) as the contrast of interest resulted in 49 DEGs (adjusted q-value <=0.05) in sgACC and 3 DEGs in Ant-Ins. Suicide completion, our mortality outcome of interest, was associated with 54 DEGs in sgACC and 6 DEGs in Ant-Ins. Longevity (i.e., another data reduction identified a factor measuring higher age at death and related variables despite chronic lifetime psychiatric and medical illnesses), on the other hand, was associated with more DEGs in Ant-Ins (145 DEGs) than in sgACC (14 DEGs).

Similar to our Ant-Ins analysis of comparing extreme scores for *psychiatric morbidity*, we assessed the sgACC transcriptome measures of DEGs by comparing 20 donors with the lowest scores on *psychiatric morbidity* vs. 20 donors with the highest scores on *psychiatric morbidity* by first including both the mood disorder and control cohorts at p<0.05 FDR. As a result, we found five downregulated and thirty-three upregulated DEGs (**Table S7A**). Then, we repeated this analysis by excluding the controls and only comparing the 20 lowest *psychiatric morbidity* scores within the mood disorder cohort vs. the 20 highest psychiatric morbidity scores. We found eighteen downregulated and 400 upregulated DEGs (**Table S7B**).

**Longevity-related differential gene expression analysis identified DEGs.**

***Longevity-associated DEGs in mood disorders and unaffected controls***: We separately assessed longevity-associated DEGs across all samples for the Ant-Ins and sgACC. We applied an identical median split-half comparison as in our *psychiatric morbidity* analysis to identify DEGs associated with high vs. low *longevity* at adjusted p<=0.05 FDR. We found that high vs. low *longevity* was associated with eighty-two downregulated Ant-Ins DEGs, including the protein synthesis *PSK5* (downregulated in Ant-Ins high *psychiatric morbidity* associated DEGs), cellular actin polymerization *ARPC5*, and RNA polymerase binding *GSG1* genes (**Table S4A; Fig S7A-B**). Conversely, we found sixty-four upregulated genes, including the glycoprotein and glycolipid synthesizer and carbohydrate metabolizer *FUK* [42], UDP-N-acetylglucosamine biosynthetic processor *UAP1L1*, and cellular calcium regulator wolframin *WFS1* genes [67,68,69,70] in the Ant-Ins (**Table S4A; Fig S7C-D**). Together, the GO-term pathways for *longevity-*associated Ant-Ins DEGs were enriched for protein synthesis, synaptic membrane, and receptor signaling genes [24,41,71]. Similar analysis of high vs. low *longevity* (including MDD, BD, and controls) in the sgACC yielded no DEGs at adjusted q-values of q<=0.01 for high vs. low *longevity* in the combined mood disorders and the unaffected controls.

***Longevity-associated DEGs in mood disorders***: We compared high vs. low *longevity* exclusively in the mood disorder samples (excluding controls) to assess DEGs for maladaptive aging, first in the Ant-Ins followed with the sgACC analysis at p<=0.05 FDR. We found one hundred and twenty-nine downregulated DEGs in the Ant-Ins, including the protein synthesis *PSK5* gene and MTCO2P1*2* pseudogene, and sixty-four upregulated genes (**Table S4A)**. Unlike the lack of DEGs in the sgACC of the combined samples of mood disorders and control, our assessment of DEGs associated with *longevity* in mood disorders only (excluding controls) yielded ten downregulated genes, including GTPase activator and cellular cytoskeletal and apoptosis regulator *ARHGAP10* gene previously implicated in brain morphogenesis and schizophrenia [72-74], and twelve upregulated genes, including protein-kinase ROCK1P1 pseudogene (**Table S4B**). The GO-terms for *longevity*-associated DEGs in sgACC identified enriched pathways for tyrosine and protein synthesis and folding, cellular apoptosis, body assembly, and negative transcriptional regulatory genes [75,76] in the mood disorders-only cohort.

***Longevity-associated DEGs validated in unaffected controls***: Unlike *psychiatric morbidity*, the presence of which was an exclusion criterion for controls, the phenotypic loading for our identified higher-order *longevity* factor (which reflects variability in *a.* marital status, *b.* # of children, *c.* Axis-III comorbidity, and *d.* age at death) was naturally expected to be more generally distributed across all samples including controls. As such, we treated variability in *longevity* in controls as a proxy measure of how socially enriched the donors’ lives were (marital status & #of children) in addition to how long they lived despite the presence of chronic medical diseases (Axis-III morbidity and age at death). With the adaptive measure of *longevity* in controls in mind, we conducted a differential gene expression analysis of high vs. low *longevity* exclusively in the unaffected controls using the p<=0.05 FDR threshold to identify DEGs associated with mentally adaptive/resilient *longevity* (i.e., having no recorded lifetime history of *psychiatric morbidity*). Comparing high vs. low adaptive *longevity* in controls only, we found thirty-four upregulated DEGs in the Ant-Ins (**Table S5A**), including the GTPase and metal iron binding gene implicated in autism AGAP7P gene, G-protein coupled receptor S1PR2, and LPAR4 genes, Leucine-rich LRRC69 gene, DNA binding transcription factor SHOX, antisense RNA WT1-AS gene, fibroblast growth factor 23 *FGF23* anti-aging gene [77], the pregnancy-specific Beta-1-Glycoprotein 2 *PSG2* [78] gene, and several other genes and pseudogenes (**Table S5A; Fig S8A**). We further found twenty-five downregulated DEGs (**Table S5A**) associated with adaptive *longevity* in controls only in Ant-Ins, including AC007192.6/PIK3R2 gene involved in neurodevelopment, and the collagen type VI alpha chain COL6A3 gene associated with connective tissue/muscle regeneration and disease (**Table S6A**), etc. Assessing the GO-terms for *longevity*-associated DEGs in Ant-Ins of controls revealed pathways enriched for cellular response to vitamin D metabolic processes, lipid metabolism, and cellular homeostasis [42,43] (**Fig S8A-B)**.

High vs. low adaptive *longevity*-associated DEGs in the sgACC of controls only identified one upregulated uncharacterized marker, STX16-NPEPL1. However, high longevity versus low longevity controls comparison in the sgACC resulted in twenty downregulated genes, including the blood leukocyte chaperoned cytokine-stimulated *SELE* and tumor necrosis factor-related apoptosis inducer *TNFRSF10A* [35,38], as well as *TMEM45B*, *SEMA3F*, *ADGRL4*, *VASP*, *SOCS3*, *ADAMTS1*, *DNAJB1*, *ICAM2*, and *NOS3* genes. Further downregulated DEGs associated with adaptive *longevity* in sgACC include the major histocompatibility complex-heat shock protein *HSPA1A* and *HSPB1*, as well as *PLA1A* and *CNN2* genes, and an uncharacterized PUDP*,* AF131216.6 genes (**Table S5B**). Additionally, these downregulated DEGs included the calcium membrane *ORAI1* that channels calcium influx into T-Cells [79-82], the T-cell-signaling G-protein-coupled prostaglandin E receptor *PTGER4* gene (Hsiao et al. 2021), and the synaptic membrane/GTPace signal transducers *PLEKHG1* genes (**Table S5B; Fig S8C-D**). GO-terms for adaptive *longevity*-associated DEGs in sgACC were enriched for tyrosine or protein synthesis/folding, apoptosis, body assembly, and negative transcriptional regulation [75,76] (**Fig S8C-D)**.

**SUPPLEMENTARY DISCUSSION**

In line with the increased risk for adverse childhood traumatic experiences coupled with the increased likelihood of socioeconomic adversity for individuals with familial risk for mood disorders, our postmortem mood disorder samples comprising 112 of the 180 donors, we found that the observed DEGs in the Ant-Ins network concerning high vs. low *longevity* in controls were associated with downregulation in protein synthesis and upregulated synaptic membrane signaling. On the one hand, our observed *longevity*-associated DEGs specific to the controls, akin to adaptive *longevity/aging*, were both upregulated in biological systems associated with enhancing anti-aging or longevity processes, as well as in cellular homeostatic processes such as response to vitamin D metabolic processes and lipid metabolism [42,43]. Adaptive *longevity*/no lifetime psychiatric disease in controls was additionally associated with downregulated DEGs related to neurodevelopment, connective tissue, and muscular degeneration, potentially reflecting the aging and physical disease-related brain molecular correlates for resilience to mood dysfunctions. On the other hand, DEGs for high vs. low adaptive *longevity* in sgACC in controls were associated with downregulated cellular apoptosis, negative regulation of protein synthesis and folding, negative regulation of stress-induced transcriptional activity, and response to heat shock.

As humans develop from birth to adulthood and gradually attain advanced aging, repeated exposures to social and environmental pathogens can negatively impact brain and body functions, leading to various maladaptive phenotypes and diseases like mood disorders. It is, therefore, plausible that the phenotypic expression of lifetime resilience to mood disorders and co-occurring chronic medical diseases is essential for the long-term maintenance of well-being and longevity. While previous studies have examined the relationship between lifetime mood disorder risks and longevity, using different metrics such as years of life lost, see an influential review on this [2], the neurobiology underlying variability in mood disorder morbidity and related mortality phenotypes remains obscure.

The findings of downregulated apoptotic and inhibitory processes for adaptive biological functions in individuals who died without ever having any known psychiatric disorders suggest a proximate role for the sgACC cellular integrity in maintaining functional behavioral health across the lifespan. Future studies will be needed to validate these region-specific findings. However, our results of adaptive *longevity*-associated upregulatory anti-aging molecular pathway functions, coupled with pregnancy-specific and cellular metabolic/homeostatic DEGs in Ant-Ins, as opposed to downregulatory DEGs related to accelerated cell death and inhibitory processes for protein synthesis in sgACC in controls with no history of psychiatric diseases, strongly point to possible adaptive molecular processes that may be necessary for the maintenance of basic cellular and neuronal regulation of complex affective behaviors in the AIAC brain network. If replicated, these observed novel mechanisms may be critical to the basic molecular regulation of longevity, even in age-related comorbid medical conditions.

**SUPPLEMENTARY FIGURES**

**Supplementary Figure 1 (Fig S1). A**, shows the targeted dissected regional locale of the ventral anterior-most portion of the Ant-Ins. Frozen tissue was dissected from the Ant-Ins section for each donor for RNA sequencing by targeting the region most well-documented to be volumetrically reduced in mood disorders. , shows the dissected regional volume from the sgACC targeting the locale of the ventral ACC Brodmann area 25 that intersects with the curvature of the Brodmann area 32.

**Figure S2**. **RRHO Enrichment Results**. The significant heatmaps illustrate quadrants (top right) or (bottom left) of hypergeometric overlap in gene expression changes in the same direction in the Ant-Ins and sgACC regions of the AIAC network. **A**) Shows that for high versus low psychiatric morbidity, there are inter-regional overlaps in both downregulated and upregulated gene expression. B) For suicide completion, however, the preponderance of overlap in Ant-Ins and sgACC regional gene expression was predominantly downregulated, similar to longevity-associated gene expression patterns observed in C. The color bars on the right represent the values of the log10 p-values of the correlations.

**Fig S3. Gene Ontology Terms Derived from WGCNA of Ant-Ins.** **A** illustrates that Axis-III comorbidity correlated negatively with the Ant-Ins tan module, which is enriched for metabolic, energy transport, and mitochondrial translation/gene co-expression genes. **B**, Age at death, Axis-I, and suicide lethality collectively correlated negatively with the yellow module capturing cellular and neuronal ion channel/calcium ion-dependent signaling and synaptic membrane gene co-expression.

**Fig S4. Gene Ontology Terms Derived from WGCNA of Ant-Ins.** **A**, shows Axis-I psychiatric comorbidity correlates positively with the brown module enriched for a wide-ranging inflammatory cytokine response, T-cell immune response, and leukocyte functions gene co-expression in the Ant-Ins. In contrast, B illustrates the positive correlations between Axis-III comorbidity and the magenta/green module enriched for biosynthesis/protein synthesis and viral transcription in Ant-Ins.

**Fig S5. Gene Ontology Terms Derived from WGCNA of sgACC.** A show that the sgACC gene expression reflects a positive correlation between Axis-I and the salmon module enriched for spliceosome, thyroid hormone, and notch signaling gene co-expression. B shows negative correlations between Axis-III comorbidity and BMI with the WGCNA tan module enriched for ribosomal, spliceosomal, mRNA transport and methylation, and protein synthesis gene co-expression in sgACC.

**Fig S6**. A shows that Axis-III comorbidity correlates negatively with the WGCNA grey module capturing cellular immune and developmental regulatory gene co-expression in the sgACC. **B** shows that the WGCNA red, pink, cyan, tan, grey, and green modules enriched for metabolic, protein synthesis, and bodily homeostatic regulatory gene co-expression in sgACC were associated with Axis-III comorbidity and BMI.

** Fig S7**. **Gene Ontology (GO) terms and Volcano plots for High vs Low Longevity (higher-order factor)**. **A**) illustrates GO terms for GECs in Ant-Ins for high longevity vs. low longevity in all samples, with **B**) depicting the related volcano plot for the Ant-Ins results in **A** for the combination of mood disorders and unaffected control samples. **C & D**) illustrates GO terms in sgACC representing high longevity vs. low longevity for all samples and related volcano plots for the sgACC results in **C**. Because the -log10 (adjusted q-values) of a large number of genes in Insula was close to zero, volcano plots were generated using q-value instead of adjusted q-value. Genes meeting the following cutoffs, adjusted q-value <0.05, and absolute log2 fold change >= 1 were highlighted on the volcano plot as significant genes.

**Fig S8**. **Gene Ontology (GO) terms and Volcano plots for High vs Low adaptive Longevity (in controls only)**. **A**) illustrates GO terms for GECs in Ant-Ins for high longevity vs. low longevity in controls, and **B**) related volcano plot for the results in **A** for unaffected controls. **C & D**) illustrates GO terms in sgACC for high adaptive longevity vs. low adaptive longevity in unaffected controls and related volcano plots in **C**. Because the -log10 (adjusted q-values) of a large number of genes in Insula was close to zero, volcano plots were generated using q-value instead of adjusted q-value. Genes meeting the following cutoffs: adjusted q-value <0.05 and absolute log2 fold change >= 1 were highlighted on the volcano plot as significant genes.

**SUPPLEMENTARY REFERENCES**

*Continuation from refs 1-66 I main manuscript body*

67. Koido K, Kõks S, Nikopensius T, Maron E, Altmäe S, Heinaste E, Vabrit K, Tammekivi V, Hallast P, Kurg A, Shlik J, Vasar V, Metspalu A, Vasar E. [Polymorphisms in wolframin (WFS1) gene are possibly related to increased risk for mood disorders.](https://pubmed.ncbi.nlm.nih.gov/15473915/) Int J Neuropsychopharmacol. 2005; 8(2): 235-44.

68. Kato T, Ishiwata M, Yamada K, Kasahara T, Kakiuchi C, Iwamoto K, Kawamura K, Ishihara H, Oka Y. [Behavioral and gene expression analyses of Wfs1 knockout mice as a possible animal model of mood disorder.](https://pubmed.ncbi.nlm.nih.gov/18343518/) Neurosci Res. 2008; 61(2): 143-58.

69. Munshani S, Ibrahim EY, Domenicano I, Ehrlich BA. The Impact of Mutations in Wolframin on Psychiatric Disorders. Front Pediatr. 2021; 9: 718132.

70. Xavier J, Bourvis N, Tanet A, Ramos T, Perisse D, Marey I, Cohen D, Consoli A. [Bipolar Disorder Type 1 in a 17-Year-Old Girl with Wolfram Syndrome.](https://pubmed.ncbi.nlm.nih.gov/27045389/) J Child Adolesc Psychopharmacol. 2016; 26(8): 750-755.

71. Szczepankiewicz D, Narożna B, Celichowski P, Sakrajda K, Kołodziejski P, Banach E, Zakowicz P, Pruszyńska-Oszmałek E, Pawlak J, Wiłkość M, Dmitrzak-Węglarz M, Skibińska M, Bejger A, Twarowska-Hauser J, Rybakowski JK, Nogowski L, Szczepankiewicz A. Genes involved in glucocorticoid receptor signaling affect susceptibility to mood disorders. World J Biol Psychiatry. 2021; 22(2): 149-160.

72. Kim J, Shim S, Choi SC, Han JK. A putative Xenopus Rho-GTPase activating protein (XrGAP) gene is expressed in the notochord and brain during early embryogenesis. Gene Expr Patterns. 2003; 3(2): 219-23.

73. Sekiguchi M, Sobue A, Kushima I, Wang C, Arioka Y, Kato H, Kodama A, Kubo H, Ito N, Sawahata M, Hada K, Ikeda R, Shinno M, Mizukoshi C, Tsujimura K, Yoshimi A, Ishizuka K, Takasaki Y, Kimura H, Xing J, Yu Y, Yamamoto M, Okada T, Shishido E, Inada T, Nakatochi M, Takano T, Kuroda K, Amano M, Aleksic B, Yamomoto T, Sakuma T, Aida T, Tanaka K, Hashimoto R, Arai M, Ikeda M, Iwata N, Shimamura T, Nagai T, Nabeshima T, Kaibuchi K, Yamada K, Mori D, Ozaki N. ARHGAP10, which encodes Rho GTPase-activating protein 10, is a novel gene for schizophrenia risk. Translational Psychiatry. 2020; 10(1): 247.

74. Hada K, Wulaer B, Nagai T, Itoh N, Sawahata M, Sobue A, Mizoguchi H, Mori D, Kushima I, Nabeshima T, Ozaki N, Yamada K. Mice carrying a schizophrenia-associated mutation of the Arhgap10 gene are vulnerable to the effects of methamphetamine treatment on cognitive function: association with striatal neuron morphological abnormalities. Mol Brain. 2021; 14: 21.

75. Jope RS. A bimodal model of the mechanism of action of lithium. Mol Psychiatry. 1999; 4(1): 21-5.

76. Albert PR, Fiori LM. Transcriptional dys-regulation in anxiety and major depression: 5-HT1A gene promoter architecture as a therapeutic opportunity. Curr Pharm Des. 2014; 20(23): 3738-50.

77. Haussler MR, Whitfield GK, Haussler CA, Sabir MS, Khan Z, Sandoval R, Jurutka PW. 1,25-Dihydroxyvitamin D and Klotho: A Tale of Two Renal Hormones Coming of Age.

Vitam Horm. 2016; 100: 165-230.

78. Khan WN, Teglund S, Bremer K, Hammarström S. The pregnancy-specific glycoprotein family of the immunoglobulin superfamily: identification of new members and estimation of family size. Genomics. 1992; 12(4): 780-7.

79. Luik RM, Lewis RS. New insights into the molecular mechanisms of store-operated Ca2+ signaling in T cells. Trends Mol Med. 2007r; 13(3): 103-7.

80. Miller AH. Depression and immunity: a role for T cells? Brain Behav Immun. 2010; 24: 1-8.

81. Ramesh G, Jarzembowski L, Schwarz Y, Poth V, Konrad M, Knapp ML, Schwär G, Lauer AA, Grimm MOW, Alansary D, Bruns D, Niemeyer BA. A short isoform of STIM1 confers frequency-dependent synaptic enhancement. Cell Rep. 2021; 34(11): 108844.

82. Voros O, Panyi G, Hajdu P. Immune Synapse Residency of Orai1 Alters Ca^2+^ Response of T Cells. Int J Mol Sci. 2021; 22(21): 11514.

# **TABLES**

**Tables 1** Gene expression differences detected at adjusted p<0.05. Gray-shaded results represent downregulated genes (negative Log2Foldchange values), whereas non-shaded results represent upregulated genes (positive Log2Foldchange values).

| **TABLE 1 Psychiatric and Chronic Disease/Medical Comorbidity Presented as % of Comorbid Axis III Diseases** | | | | | | | | | | |
| --- | --- | --- | --- | --- | --- | --- | --- | --- | --- | --- |
| **Primary Axis I Diagnosis** | Lung | Cardiovascular | Cancer | Diabetes/Endocrine | Inflammatory/ Chronic Pain | Subst. Intoxication/Poisoning | Other CNS | Infection | Obesity (BMI>=25) | # of Donors |
| Bipolar Disorder (BD) | 17.30% | 36.50% | 9.60% | 19.3%% | 15.40% | 42.30% | 19.20% | 5.80% | 71% | 52 |
| Major Depressive Disorder (MDD) | 13.30% | 50.00% | 5.00% | 16.7%% | 5.00% | 46.70% | 8.30% | 5.00% | 59.26% | 60 |
| Unaffected controls | 8.80% | 74.40% | 7.40% | 13.2%% | 4.40% | 0.00% | 2.90% | 2.90% | 81.66% | 68 |
| Abbreviations: Lung = lung disease; Endocri. = Endocrine diseases/obesity; Subst. = Substance; CNS = Central Nervous System Diseases such as migraine or epilepsy with no focal localization in the AIAC network; # = number | | | | | | Total # of Unique Donors | | | | 180 |

**SUPPLEMENTARY TABLES 1-7**

In the supplementary tables below, gray-shaded results represent downregulated genes (negative Log2Foldchange values), whereas non-shaded results represent upregulated genes (positive Log2Foldchange values).

| **TABLE S1** | | |
| --- | --- | --- |
| **Table S1A** | | |
| Major Depression > Controls in Ant-Ins | | |
| GeneName | log2foldchange | q-adjusted |
| SELE | -2.57 | 0.041 |
| H19 | 1.82 | 0.003 |
| MTCO2P12 | 2.55 | 0.006 |
| FBXO47 | 3.00 | 0.006 |
| IL1RL1 | 2.61 | 0.025 |
| **Table S1B** | | |
| Bipolar disorder > Controls in Ant-Ins | | |
| GeneName | Log2FoldChange | q-adjusted |
| MTND1P23 | -3.28 | 7.92E-05 |
| RP11-352E6.2 | -2.79 | 0.0177 |
| RP1-193H18.3 | 2.01 | 0.0017 |
| CTD-2336O2.3 | 0.48 | 0.0194 |

| **TABLE S2A** | | | |
| --- | --- | --- | --- |
| *Psychiatric Morbidity* in Ant-Ins (Mood Disorders & Controls) | | | |
| GeneName | | log2FoldChange | q-adjusted |
| PCSK5 | | -3.46 | 1.61E-07 |
| HSPA7 | | -2.74 | 1.54E-06 |
| CCL4 | | -1.87 | 0.000708 |
|  | |  |  |
| *Psychiatric Morbidity* in Ant-Ins (Mood Disorders Only) | | | |
| GeneName | | log2FoldChange | q-adjusted |
| PCSK5 | | -2.97 | 0.02978 |
| HSPA7 | | -2.36 | 0.04995 |
| MTCO1P12 | | -1.99 | 0.04995 |
|  | |  |  |
| **Table S2B** | | | |
| *Psychiatric morbidity* in sgACC (Mood Disorders & Controls) | | | |
| GeneName | log2FoldChange | | q-adjusted |
| SORD2P | -0.96 | | 3.46E-05 |
| MTRNR2L12 | -1.31 | | 0.0002 |
| HMGB1P5 | -1.21 | | 0.027 |
| LGR6 | -0.82 | | 0.0001 |
| NTRK1 | -0.92 | | 0.027 |
| FCGBP | -0.68 | | 0.0131 |
| USP51 | -0.17 | | 0.0316 |
| HKDC1 | -0.54 | | 0.041 |
| DOLK | -0.16 | | 0.0428 |
| CEP41 | -0.12 | | 0.0428 |
| ZNF252P | -0.12 | | 0.0466 |
| C1orf194 | -0.6 | | 0.0492 |
| HILPDA | 1.38 | | 5.81E-07 |
| IL1RL1 | 1.07 | | 1.73E-05 |
| SFN | 1.23 | | 0.0001 |
| MT1X | 0.88 | | 0.0015 |
| CH507-513H4.3 | 1.3 | | 0.0038 |
| CH507-513H4.4 | 1.3 | | 0.0038 |
| CH507-513H4.6 | 1.3 | | 0.0038 |
| C3orf20 | 0.45 | | 0.0078 |
| CDH3 | 0.26 | | 0.0078 |
| HAMP | 0.69 | | 0.0088 |
| SMTNL1 | 0.36 | | 0.0107 |
| ADAMTS9-AS1 | 0.4 | | 0.0121 |
| MTCO3P12 | 0.8 | | 0.0131 |
| VEGFA | 0.59 | | 0.0158 |
| MT1M | 0.6 | | 0.0165 |
| FAM101B | 0.33 | | 0.0168 |
| ANGPTL4 | 0.72 | | 0.027 |
| CTAGE6 | 0.74 | | 0.027 |
| GNA14 | 0.36 | | 0.027 |
| LURAP1L | 0.35 | | 0.0273 |
| OSMR | 0.6 | | 0.027 |
| PLGLB1 | 0.19 | | 0.027 |
| RRN3P1 | 0.13 | | 0.027 |
| ADAMTS9 | 0.55 | | 0.0271 |
| BCRP3 | 0.4 | | 0.0282 |
| RP11-61L23.2 | 0.45 | | 0.0282 |
| SERPINA3 | 1.37 | | 0.0282 |
| GRHL3 | 0.98 | | 0.0401 |
| SOCS3 | 1.21 | | 0.0428 |
| ZNF587 | 0.09 | | 0.0428 |
| GADD45A | 0.47 | | 0.0466 |
| KRT18P7 | 0.25 | | 0.0466 |
| PRELID2 | 0.2 | | 0.0492 |
| RP11-1277A3.2 | 0.18 | | 0.0492 |
| RP13-20L14.10 | 0.22 | | 0.0492 |

| **TABLE S3**  **TABLE S3A** | | |
| --- | --- | --- |
| Suicide Completion in Ant-Ins (Mood Disorders Only) | | |
| GeneName | log2FoldChange | q-adjusted |
| BAALC-AS1 | -1.39 | 0.0010 |
| FOSB | -2.11 | 0.0054 |
| SERPINA3 | -3.24 | 0.0054 |
| CHI3L1 | -1.82 | 0.0065 |
| AC145676.2 | -0.91 | 0.0084 |
| SLC39A14 | -0.89 | 0.0442 |
| **Table S3B** | | |
| Suicide completion in sgACC (Mood Disorders Only) | | |
| GeneName | log2FoldChange | q-adjusted |
| RP11-403A3.3 | -1.86 | 5.66E-07 |
| SERPINA3 | -3.24 | 5.66E-07 |
| CHI3L1 | -1.92 | 9.00E-07 |
| SFN | -2.14 | 3.62E-06 |
| SLC11A1 | -1.32 | 6.00E-06 |
| ICAM1 | -1.31 | 7.63E-06 |
| GBP2 | -1.26 | 1.27E-05 |
| TNFRSF1A | -0.82 | 1.37E-05 |
| BAG3 | -1.42 | 2.20E-05 |
| F3 | -1.04 | 2.20E-05 |
| IL1RL1 | -1.74 | 2.84E-05 |
| BAALC-AS1 | -0.75 | 6.25E-05 |
| HILPDA | -1.96 | 6.25E-05 |
| OSMR | -1.27 | 0.000101 |
| EMP1 | -1.38 | 0.000103 |
| KIAA0040 | -1.28 | 0.000106 |
| MTHFD2 | -0.81 | 0.000108 |
| PDPN | -1.07 | 0.000111 |
| SLC39A14 | -0.98 | 0.000111 |
| C1R | -0.78 | 0.000125 |
| SLC44A3 | -0.62 | 0.000125 |
| MYO1G | -1.23 | 0.000146 |
| MAP3K6 | -0.66 | 0.000156 |
| SIX4 | -0.96 | 0.000166 |
| SERPINA1 | -1.58 | 0.000175 |
| SERPINE1 | -1.10 | 0.000175 |
| MT1X | -1.50 | 0.000213 |
| RP11-473M20.16 | -1.26 | 0.000244 |
| HAMP | -1.25 | 0.000353 |
| SERPINH1 | -1.71 | 0.000353 |
| TNFRSF12A | -1.26 | 0.000405 |
| MYC | -0.94 | 0.000421 |
| FUT3 | -0.69 | 0.000431 |
| MGST1 | -0.45 | 0.000433 |
| TEAD3 | -0.76 | 0.000444 |
| CEBPD | -1.15 | 0.000501 |
| FPR1 | -1.14 | 0.000501 |
| GBP1 | -1.10 | 0.000501 |
| IL1R1 | -1.20 | 0.000501 |
| LINC01057 | -0.48 | 0.000564 |
| C1S | -0.59 | 0.000564 |
| CNN3 | -0.60 | 0.000564 |
| FGF2 | -0.68 | 0.000564 |
| HSPA7 | -1.70 | 0.000564 |
| DTNA | -0.55 | 0.000628 |
| BCL3 | -0.90 | 0.000658 |
| PLSCR1 | -0.87 | 0.000658 |
| SMAD1 | -0.25 | 0.000658 |
| DENND2D | -0.95 | 0.000747 |
| TPST1 | -0.49 | 0.000752 |
| CDKN1A | -1.43 | 0.000885 |
| ANGPTL4 | -1.36 | 0.00092 |
| CD163 | -0.67 | 0.000957 |
| LIMK2 | -0.64 | 0.001048 |
| YTHDF1 | -0.38 | 0.001234 |
| PNLDC1 | -1.07 | 0.001244 |
| ACTRT3 | -0.95 | 0.001422 |
| APOL6 | -0.38 | 0.001422 |
| IFITM2 | -1.17 | 0.001422 |
| LGALS3 | -0.53 | 0.001422 |
| TLR2 | -0.87 | 0.001422 |
| C1RL | -0.64 | 0.001448 |
| HMOX1 | -0.79 | 0.001448 |
| IL4R | -0.94 | 0.001448 |
| CD93 | -1.06 | 0.001473 |
| FCGR3A | -1.26 | 0.001528 |
| RP11-50D9.3 | -0.85 | 0.00186 |
| CEBPB | -0.51 | 0.001913 |
| CD14 | -1.08 | 0.001999 |
| PSTPIP2 | -0.65 | 0.0023 |
| A4GALT | -0.89 | 0.0023 |
| SOD2 | -0.35 | 0.002545 |
| PDLIM4 | -1.05 | 0.002983 |
| YBX3 | -0.82 | 0.002983 |
| GADD45A | -0.88 | 0.003079 |
| PIM1 | -0.65 | 0.003289 |
| ANO6 | -0.44 | 0.003376 |
| PLAC4 | -0.75 | 0.003639 |
| FCGR1A | -1.01 | 0.00367 |
| ADM | -1.78 | 0.003917 |
| MAOB | -0.38 | 0.003917 |
| ITPKC | -0.54 | 0.00415 |
| NAMPT | -0.55 | 0.004237 |
| BACE2 | -0.63 | 0.004328 |
| FERMT3 | -0.71 | 0.004588 |
| TIFA | -0.44 | 0.004588 |
| STOM | -0.46 | 0.004769 |
| C10orf10 | -1.10 | 0.004809 |
| GLIS3 | -0.62 | 0.004809 |
| MIR548AJ2 | -0.41 | 0.004809 |
| MT2A | -0.78 | 0.004809 |
| RP11-482G13.1 | -0.82 | 0.004809 |
| SBNO2 | -0.75 | 0.004819 |
| EMILIN2 | -0.65 | 0.004836 |
| OSMR-AS1 | -0.52 | 0.004836 |
| TUBB6 | -0.72 | 0.004997 |
| IFITM3 | -0.92 | 0.005059 |
| JAK3 | -0.55 | 0.005409 |
| SERPING1 | -0.58 | 0.005416 |
| ASAP3 | -0.39 | 0.005866 |
| PTPN2 | -0.19 | 0.005886 |
| RDH10 | -0.70 | 0.005886 |
| SORBS1 | -0.30 | 0.005886 |
| CP | -1.40 | 0.005896 |
| EMP3 | -0.60 | 0.006086 |
| ETV6 | -0.34 | 0.006086 |
| IFITM1 | -0.88 | 0.006086 |
| SOCS3 | -2.02 | 0.006274 |
| SLCO4A1 | -0.90 | 0.006389 |
| FAS | -0.56 | 0.006756 |
| HIF1A | -0.29 | 0.006981 |
| CLU | -0.35 | 0.007428 |
| CLEC2B | -0.54 | 0.007466 |
| RP11-211G3.2 | -0.87 | 0.007466 |
| STC1 | -1.04 | 0.007466 |
| CFI | -0.78 | 0.007491 |
| RP11-403A3.2 | -0.60 | 0.007537 |
| PLOD2 | -0.47 | 0.007671 |
| LSM6 | -0.26 | 0.007728 |
| SIGLEC9 | -0.59 | 0.007815 |
| AL773572.7 | -0.87 | 0.007954 |
| ALOXE3 | -1.00 | 0.008049 |
| CDK2 | -0.86 | 0.008049 |
| HSPB1 | -0.92 | 0.008049 |
| SLC16A3 | -0.74 | 0.008774 |
| GBP3 | -0.72 | 0.008781 |
| TMEM176B | -0.60 | 0.008936 |
| CTAGE6 | -1.09 | 0.009065 |
| ADAMTS9 | -0.95 | 0.00919 |
| CD59 | -0.26 | 0.009565 |
| PRRX1 | -0.39 | 0.009737 |
| AHCTF1 | -0.19 | 0.010316 |
| APOL4 | -0.30 | 0.01055 |
| G0S2 | -0.85 | 0.01055 |
| PLSCR4 | -0.43 | 0.01055 |
| TIPARP | -0.72 | 0.01055 |
| TRAF3IP2 | -0.31 | 0.01055 |
| STC2 | -1.19 | 0.010741 |
| CD44 | -1.32 | 0.010799 |
| TUBA1C | -0.48 | 0.010799 |
| MAN1C1 | -0.36 | 0.010991 |
| ZNF436-AS1 | -0.27 | 0.01116 |
| DSC2 | -0.39 | 0.0115 |
| LILRB3 | -0.41 | 0.011872 |
| RP11-106M7.1 | -0.42 | 0.011873 |
| FSTL1 | -0.54 | 0.011994 |
| MRVI1 | -0.52 | 0.012286 |
| BOC | -0.53 | 0.012413 |
| CASP1 | -0.72 | 0.012413 |
| PGAM2 | -1.25 | 0.012413 |
| STON1 | -0.88 | 0.012413 |
| TIMP1 | -0.63 | 0.012413 |
| ARID5A | -0.62 | 0.01257 |
| GNA14 | -0.56 | 0.01257 |
| NNMT | -0.95 | 0.01257 |
| RFX4 | -0.50 | 0.01257 |
| DSE | -0.32 | 0.012607 |
| GLRX | -0.45 | 0.012976 |
| IFI30 | -0.88 | 0.01324 |
| ADIRF-AS1 | -0.35 | 0.014055 |
| NFKB2 | -0.58 | 0.01449 |
| STEAP3 | -0.69 | 0.014582 |
| CISH | -1.18 | 0.014583 |
| MS4A6A | -0.39 | 0.014583 |
| RP11-274H2.5 | -0.75 | 0.014583 |
| SLCO4A1-AS1 | -0.78 | 0.015076 |
| TMBIM1 | -0.65 | 0.015981 |
| CRISPLD1 | -0.73 | 0.016063 |
| RP11-572O17.1 | -0.91 | 0.016346 |
| LINC01480 | -0.30 | 0.016437 |
| C2 | -0.37 | 0.016501 |
| PTPN22 | -0.36 | 0.016501 |
| RP13-20L14.6 | -0.46 | 0.016501 |
| RAB20 | -0.82 | 0.016549 |
| MR1 | -0.21 | 0.016807 |
| MRVI1-AS1 | -0.49 | 0.016807 |
| RAB13 | -0.47 | 0.017828 |
| STAT3 | -0.37 | 0.017828 |
| EZH2 | -0.28 | 0.01804 |
| AHCYL1 | -0.37 | 0.018132 |
| C1QTNF1 | -0.50 | 0.018132 |
| COL4A1 | -0.68 | 0.018132 |
| MFAP5 | -0.61 | 0.018132 |
| TRAF6 | -0.15 | 0.018132 |
| FCGR2A | -0.93 | 0.018167 |
| KCNE4 | -0.24 | 0.018167 |
| BMPR1A | -0.21 | 0.018179 |
| RDH10-AS1 | -0.92 | 0.01819 |
| THBD | -0.85 | 0.018724 |
| CASP7 | -0.46 | 0.01921 |
| SIGLEC14 | -0.76 | 0.019898 |
| PPP1R3B | -0.69 | 0.020232 |
| RP11-50D9.4 | -0.81 | 0.020365 |
| MT1M | -0.90 | 0.021105 |
| NOD1 | -0.43 | 0.021105 |
| RP11-304F15.3 | -0.43 | 0.021105 |
| SLC25A37 | -0.28 | 0.021105 |
| TNFRSF10D | -0.67 | 0.021105 |
| PHACTR2P1 | -0.56 | 0.021385 |
| SLC17A9 | -0.52 | 0.021577 |
| WWTR1 | -0.67 | 0.021613 |
| GPR4 | -0.58 | 0.021636 |
| MFHAS1 | -0.26 | 0.021636 |
| RP11-70J12.1 | -0.70 | 0.021636 |
| PPP1R18 | -0.48 | 0.021767 |
| LINC00869 | -0.18 | 0.022252 |
| NUPR1 | -0.46 | 0.022252 |
| SMTNL1 | -0.47 | 0.023367 |
| UNG | -0.21 | 0.023444 |
| ALPK1 | -0.23 | 0.023503 |
| TEAD2 | -0.63 | 0.023503 |
| ATXN7 | -0.14 | 0.024555 |
| AC063976.7 | -0.53 | 0.024808 |
| EPHA2 | -0.81 | 0.024808 |
| PPRC1 | -0.18 | 0.024808 |
| PYGL | -0.52 | 0.024808 |
| RHPN2 | -0.37 | 0.024808 |
| LTF | -1.53 | 0.024876 |
| TNFSF13B | -0.40 | 0.024876 |
| FAM20C | -0.46 | 0.024904 |
| THBS1 | -0.67 | 0.024904 |
| LINC01532 | -0.64 | 0.025127 |
| PDLIM1 | -0.83 | 0.02547 |
| IER5L | -0.52 | 0.025545 |
| AEBP1 | -0.87 | 0.025662 |
| AQP4 | -0.52 | 0.025662 |
| ASPH | -0.20 | 0.025662 |
| DTX3L | -0.42 | 0.025662 |
| NSMAF | -0.13 | 0.025662 |
| PNRC1 | -0.23 | 0.025662 |
| RGS16 | -0.81 | 0.025662 |
| RP11-244H3.1 | -0.35 | 0.025662 |
| SQRDL | -0.48 | 0.025662 |
| TGM2 | -0.70 | 0.025662 |
| TAP1 | -0.52 | 0.025688 |
| RHOC | -0.37 | 0.026709 |
| CSTB | -0.19 | 0.026831 |
| IL1B | -1.26 | 0.026831 |
| DDIT4L | -0.58 | 0.027646 |
| RIN3 | -0.37 | 0.028413 |
| ZFP36 | -1.33 | 0.028823 |
| MYZAP | -0.76 | 0.029168 |
| ARHGEF35 | -0.68 | 0.029223 |
| SELP | -0.63 | 0.029398 |
| C4A-AS1 | -0.56 | 0.029625 |
| C4B-AS1 | -0.56 | 0.029625 |
| SPHK1 | -0.64 | 0.029645 |
| RP11-274H2.2 | -0.32 | 0.029979 |
| ATP6V1B1 | -0.71 | 0.030165 |
| S100A10 | -0.74 | 0.030165 |
| HLA-F-AS1 | -0.33 | 0.030194 |
| IL15RA | -0.64 | 0.030194 |
| IL1RAP | -0.23 | 0.030345 |
| DNAJB1 | -0.87 | 0.030395 |
| RP11-122G18.12 | -0.69 | 0.0306 |
| BHLHE40 | -0.38 | 0.030667 |
| P4HA2 | -0.37 | 0.030667 |
| TMEM132E | -0.49 | 0.030667 |
| TYMP | -0.58 | 0.030667 |
| FEM1C | -0.27 | 0.031134 |
| CCND2-AS1 | -0.63 | 0.031309 |
| POM121L9P | -0.67 | 0.031559 |
| RELL1 | -0.49 | 0.031743 |
| S100A11 | -0.73 | 0.031935 |
| ANXA2 | -0.65 | 0.031942 |
| LINC00963 | -0.24 | 0.031942 |
| MPZL2 | -0.65 | 0.032269 |
| STEAP4 | -0.28 | 0.032458 |
| SEMA4B | -0.26 | 0.03252 |
| VEGFA | -0.85 | 0.033613 |
| RP1-111D6.3 | -0.92 | 0.033864 |
| IFNLR1 | -0.58 | 0.034401 |
| TM4SF1 | -0.81 | 0.034401 |
| CD247 | -0.38 | 0.035039 |
| NAV2 | -0.27 | 0.035039 |
| CTC-444N24.8 | -0.28 | 0.035165 |
| TMEM176A | -0.44 | 0.035544 |
| VASN | -0.51 | 0.035544 |
| RBM27 | -0.12 | 0.035938 |
| ACSS3 | -0.26 | 0.03604 |
| ZNF292 | -0.10 | 0.036071 |
| RP11-798K3.2 | -0.44 | 0.036773 |
| GJA4 | -0.51 | 0.037179 |
| EPHA1-AS1 | -0.45 | 0.037407 |
| DDIT4 | -0.72 | 0.038126 |
| TCAF2 | -0.31 | 0.038126 |
| PDZK1 | -0.34 | 0.03836 |
| RUNX1 | -0.41 | 0.03836 |
| PML | -0.31 | 0.038397 |
| SEC24A | -0.11 | 0.039171 |
| RAC2 | -0.61 | 0.039633 |
| CHSY1 | -0.26 | 0.042002 |
| NEAT1 | -0.84 | 0.042297 |
| TGFB2 | -0.37 | 0.042408 |
| SLC10A1 | -0.62 | 0.04318 |
| RP11-274H2.3 | -0.42 | 0.043428 |
| FABP5 | -0.35 | 0.043496 |
| SYNM | -0.33 | 0.043496 |
| CASP4 | -0.41 | 0.043729 |
| S1PR3 | -0.31 | 0.043729 |
| PIRT | -0.95 | 0.0441 |
| RP11-61L23.2 | -0.66 | 0.044521 |
| BMPR1B | -0.37 | 0.044952 |
| GFPT2 | -0.28 | 0.044952 |
| GADD45G | -0.77 | 0.045023 |
| EDN1 | -0.69 | 0.047743 |
| MDK | -0.41 | 0.047762 |
| MAP3K14-AS1 | -0.23 | 0.048166 |
| TRIP10 | -0.69 | 0.048203 |
| HSD3B7 | -0.27 | 0.048524 |
| MAFF | -0.61 | 0.048525 |
| ANP32E | -0.20 | 0.048776 |
| FAM157A | -0.64 | 0.048776 |
| LINC00996 | 0.57 | 0.00092 |
| NAA40 | 0.15 | 0.005409 |
| KLHL3 | 0.16 | 0.006086 |
| NPAS4 | 1.96 | 0.007728 |
| COL22A1 | 0.52 | 0.010622 |
| RERGL | 0.81 | 0.01223 |
| NUDT16L1 | 0.21 | 0.012413 |
| ST8SIA2 | 0.76 | 0.013765 |
| RNU4-1 | 0.52 | 0.018179 |
| EDN3 | 0.65 | 0.01921 |
| AC005592.2 | 0.32 | 0.023367 |
| BDNF | 0.56 | 0.030194 |
| SMUG1 | 0.2 | 0.030395 |
| ABCG2 | 0.61 | 0.032191 |
| CRHBP | 0.51 | 0.032558 |
| KIRREL3-AS2 | 0.36 | 0.039855 |
| FAM13B | 0.16 | 0.043496 |
| SPRY4 | 0.33 | 0.044314 |
| CRH | 0.79 | 0.044532 |

| **TABLE S4** | | |  |
| --- | --- | --- | --- |
| **Table S4A** | | |  |
| Longevity in Ant-Ins (Mood Disorders & Controls) | | |  |
| GeneName | log2FoldChange | q-adjusted |  |
| MTND1P23 | -2.89 | 1.20E-06 |  |
| ARPC5 | -0.19 | 0.000526 |  |
| GSG1 | -0.39 | 0.000723 |  |
| TCEB1 | -0.18 | 0.008443 |  |
| RP11-1263C18.1 | -0.23 | 0.009502 |  |
| HSBP1 | -0.19 | 0.013489 |  |
| NDUFS4 | -0.22 | 0.016313 |  |
| SET | -0.14 | 0.016313 |  |
| METTL9 | -0.12 | 0.017446 |  |
| LINC01102 | -0.26 | 0.017512 |  |
| LMO4 | -0.24 | 0.018691 |  |
| MSANTD3-TMEFF1 | -0.24 | 0.021389 |  |
| CXADR | -0.23 | 0.022835 |  |
| CRK | -0.11 | 0.023006 |  |
| ARPC2 | -0.17 | 0.028745 |  |
| CHCHD3 | -0.13 | 0.028745 |  |
| PDCD2 | -0.12 | 0.028745 |  |
| RP11-271F18.4 | -0.28 | 0.028745 |  |
| SMIM10L1 | -0.19 | 0.028745 |  |
| PRKRA | -0.12 | 0.028935 |  |
| RALA | -0.13 | 0.028935 |  |
| DLGAP1-AS4 | -0.36 | 0.032106 |  |
| B3GNT2 | -0.26 | 0.032687 |  |
| DR1 | -0.17 | 0.032687 |  |
| FAM19A1 | -0.32 | 0.032687 |  |
| GVINP1 | -0.41 | 0.032687 |  |
| ST8SIA2 | -0.53 | 0.032687 |  |
| XRCC3 | 0.25 | 0.032687 |  |
| NDUFB6 | -0.19 | 0.033656 |  |
| USMG5 | -0.26 | 0.033656 |  |
| RP11-563K23.1 | -0.29 | 0.034034 |  |
| AARD | -0.39 | 0.03665 |  |
| BEX5 | -0.23 | 0.03665 |  |
| BZW1 | -0.13 | 0.03665 |  |
| C19orf81 | -0.40 | 0.03665 |  |
| CDC42 | -0.17 | 0.03665 |  |
| FRMPD2 | -0.43 | 0.03665 |  |
| NRBF2 | -0.18 | 0.03665 |  |
| SDCBP | -0.11 | 0.03665 |  |
| SMIM8 | -0.15 | 0.03665 |  |
| TTR | -1.38 | 0.03665 |  |
| UBE2F | -0.16 | 0.03665 |  |
| VPS29 | -0.14 | 0.03665 |  |
| BMP2 | -0.32 | 0.038298 |  |
| FRG1 | -0.13 | 0.038298 |  |
| RPS29 | -0.27 | 0.038298 |  |
| SDHAF3 | -0.18 | 0.038298 |  |
| UBXN2B | -0.17 | 0.038298 |  |
| PSMA2 | -0.14 | 0.039923 |  |
| CASC15 | -0.19 | 0.03998 |  |
| ADRA2A | -0.35 | 0.040463 |  |
| MYL12B | -0.18 | 0.040463 |  |
| STMN2 | -0.31 | 0.040463 |  |
| BLMH | -0.11 | 0.041902 |  |
| LSM3 | -0.19 | 0.042531 |  |
| PRPS2 | -0.27 | 0.042531 |  |
| ARNTL | -0.18 | 0.043187 |  |
| C8orf34 | -0.20 | 0.043187 |  |
| CCDC117 | -0.15 | 0.043187 |  |
| COX7C | -0.22 | 0.043187 |  |
| PCDHB2 | -0.29 | 0.043187 |  |
| PPEF1 | -0.32 | 0.043187 |  |
| SELT | -0.10 | 0.043187 |  |
| SIAH2 | -0.18 | 0.043187 |  |
| SLN | -0.61 | 0.043187 |  |
| EIF2S2 | -0.14 | 0.043683 |  |
| PTENP1 | -0.22 | 0.044001 |  |
| SERBP1 | -0.10 | 0.044001 |  |
| UBE2B | -0.14 | 0.044643 |  |
| DYRK2 | -0.25 | 0.046158 |  |
| C7orf25 | -0.30 | 0.046343 |  |
| MARCH1 | -0.23 | 0.046363 |  |
| ATP5E | -0.23 | 0.046516 |  |
| ATP5J | -0.20 | 0.046516 |  |
| CCL2 | -1.35 | 0.046516 |  |
| BZW2 | -0.20 | 0.048205 |  |
| CCDC167 | -0.22 | 0.048773 |  |
| KLRK1 | -0.51 | 0.048773 |  |
| ATP5I | -0.32 | 0.049094 |  |
| GMEB1 | -0.10 | 0.049094 |  |
| MMADHC | -0.14 | 0.049094 |  |
| TRBC2 | -0.54 | 0.049094 |  |
| FUK | 0.22 | 0.001333 |  |
| UAP1L1 | 0.30 | 0.002421 |  |
| UAP1L1 | 0.30 | 0.002421 |  |
| WFS1 | 0.37 | 0.008443 |  |
| WFS1 | 0.37 | 0.008443 |  |
| LTBP3 | 0.28 | 0.009502 |  |
| NUMA1 | 0.16 | 0.013489 |  |
| IQCA1 | 0.32 | 0.014883 |  |
| SPPL2B | 0.19 | 0.016313 |  |
| NAGLU | 0.25 | 0.017446 |  |
| AGFG2 | 0.21 | 0.020314 |  |
| TAF6L | 0.19 | 0.023006 |  |
| RHCG | 0.28 | 0.024415 |  |
| CFAP70 | 0.34 | 0.028745 |  |
| TMPRSS5 | 0.46 | 0.028745 |  |
| PAQR7 | 0.21 | 0.028935 |  |
| PIDD1 | 0.20 | 0.034034 |  |
| CPSF7 | 0.08 | 0.03665 |  |
| DNASE2 | 0.24 | 0.03665 |  |
| GREB1 | 0.19 | 0.03665 |  |
| IGDCC4 | 0.44 | 0.03665 |  |
| OPLAH | 0.48 | 0.03665 |  |
| PC | 0.22 | 0.03665 |  |
| PCDHGB1 | 0.44 | 0.03665 |  |
| PCSK4 | 0.30 | 0.03665 |  |
| PLA2G6 | 0.17 | 0.03665 |  |
| SGSH | 0.23 | 0.03665 |  |
| SSH3 | 0.22 | 0.03665 |  |
| WDR81 | 0.19 | 0.03665 |  |
| BRAT1 | 0.19 | 0.038298 |  |
| MEGF6 | 0.41 | 0.038298 |  |
| PCDHGA2 | 0.51 | 0.038298 |  |
| PLXNB2 | 0.23 | 0.038298 |  |
| RECQL5 | 0.11 | 0.039439 |  |
| ACCS | 0.30 | 0.040271 |  |
| VWCE | 0.23 | 0.040271 |  |
| SLC34A3 | 0.40 | 0.040395 |  |
| TMEM79 | 0.25 | 0.040463 |  |
| ITGB4 | 0.72 | 0.041902 |  |
| XYLT2 | 0.16 | 0.041902 |  |
| FAM90A1 | 0.26 | 0.041957 |  |
| DPP7 | 0.20 | 0.042531 |  |
| PQLC2 | 0.17 | 0.042531 |  |
| PYGB | 0.17 | 0.042531 |  |
| UNG | 0.28 | 0.042531 |  |
| IVD | 0.12 | 0.043187 |  |
| KCNN3 | 0.40 | 0.043187 |  |
| PLEC | 0.19 | 0.043187 |  |
| PARP3 | 0.15 | 0.043398 |  |
| ING5 | 0.10 | 0.043482 |  |
| GYS1 | 0.21 | 0.044001 |  |
| LINC00499 | 0.49 | 0.044001 |  |
| GMPR | 0.52 | 0.046363 |  |
| TFAP4 | 0.15 | 0.046363 |  |
| TPD52L2 | 0.09 | 0.046363 |  |
| CRB2 | 0.45 | 0.046516 |  |
| UNC93B1 | 0.36 | 0.046516 |  |
| CNNM3 | 0.14 | 0.048773 |  |
| CRYL1 | 0.25 | 0.048773 |  |
| KLHL21 | 0.18 | 0.048773 |  |
| KDM3A | 0.15 | 0.048944 |  |
| LFNG | 0.54 | 0.049094 |  |
| TPCN1 | 0.30 | 0.049094 |  |
| SLC4A11 | 0.48 | 0.049783 |  |
| Longevity in Ant-Ins (Mood Disorders only) | | |  |
| GeneName | log2FoldChange | q-adjusted |  |
| IQCA1 | -0.43 | 0.0007 |  |
| C4B | -0.98 | 0.0157 |  |
| GMPR | -0.78 | 0.0157 |  |
| WFS1 | -0.44 | 0.0157 |  |
| IGDCC4 | -0.56 | 0.0162 |  |
| TMPRSS5 | -0.58 | 0.0162 |  |
| SLC46A1 | -0.31 | 0.0172 |  |
| IGSF11 | -0.31 | 0.0174 |  |
| CCDC40 | -0.31 | 0.0176 |  |
| KDM3A | -0.23 | 0.0176 |  |
| HPR | -0.87 | 0.0192 |  |
| RHCG | -0.36 | 0.0192 |  |
| TPP1 | -0.48 | 0.0192 |  |
| BBS2 | -0.32 | 0.0202 |  |
| CTD-2353F22.1 | -1.25 | 0.0202 |  |
| LHFPL1 | -0.93 | 0.0202 |  |
| LINC00092 | -0.54 | 0.0202 |  |
| UNG | -0.39 | 0.0202 |  |
| CLU | -0.47 | 0.0204 |  |
| KCNN3 | -0.55 | 0.0204 |  |
| CFAP70 | -0.42 | 0.0209 |  |
| ACCS | -0.39 | 0.0212 |  |
| FUK | -0.22 | 0.0212 |  |
| GPR143 | -0.69 | 0.0212 |  |
| AGFG2 | -0.26 | 0.0214 |  |
| CTSH | -0.63 | 0.0234 |  |
| DNAH7 | -0.37 | 0.0234 |  |
| OPLAH | -0.62 | 0.0234 |  |
| LPIN1 | -0.21 | 0.0242 |  |
| DOCK7 | -0.32 | 0.0247 |  |
| LPP-AS2 | -0.48 | 0.0247 |  |
| H6PD | -0.31 | 0.0262 |  |
| TSPAN33 | -0.22 | 0.0262 |  |
| PLK5 | -0.42 | 0.0268 |  |
| RP5-858L17.1 | -0.34 | 0.0268 |  |
| MCCC2 | -0.24 | 0.0277 |  |
| DHX58 | -0.35 | 0.0288 |  |
| AC005336.4 | -0.96 | 0.0291 |  |
| PLIN4 | -0.97 | 0.0291 |  |
| C4A | -0.88 | 0.0291 |  |
| CD109 | -0.46 | 0.0291 |  |
| CTC-498M16.2 | -0.50 | 0.0291 |  |
| GYS1 | -0.28 | 0.0291 |  |
| PAMR1 | -0.62 | 0.0291 |  |
| PIK3IP1 | -0.33 | 0.0291 |  |
| ZNRF3 | -0.64 | 0.0291 |  |
| MIR3622A | -0.54 | 0.0303 |  |
| OSBPL11 | -0.43 | 0.0303 |  |
| PCDHGA2 | -0.63 | 0.0311 |  |
| LINC00499 | -0.62 | 0.0311 |  |
| DNASE2 | -0.30 | 0.0314 |  |
| IVD | -0.15 | 0.0314 |  |
| PHYHD1 | -0.61 | 0.0314 |  |
| RP11-159D12.2 | -0.27 | 0.0314 |  |
| RP11-437J2.3 | -0.38 | 0.0314 |  |
| SPARCL1 | -0.35 | 0.0314 |  |
| CPSF7 | -0.10 | 0.0329 |  |
| UAP1L1 | -0.29 | 0.0329 |  |
| NTRK2 | -0.46 | 0.0345 |  |
| AC069368.3 | -0.75 | 0.0358 |  |
| BMPR1B | -0.70 | 0.0361 |  |
| HVCN1 | -0.47 | 0.0361 |  |
| ACOX1 | -0.25 | 0.0363 |  |
| UNC93B1 | -0.43 | 0.0363 |  |
| ACACB | -0.57 | 0.0364 |  |
| ITGB4 | -0.89 | 0.0364 |  |
| SLC22A5 | -0.30 | 0.0364 |  |
| BMP2K | -0.40 | 0.0366 |  |
| SEMA4B | -0.41 | 0.0377 |  |
| NUMA1 | -0.18 | 0.0381 |  |
| LGI4 | -0.32 | 0.0384 |  |
| RP11-806O11.1 | -0.61 | 0.0387 |  |
| NOTCH2NL | -0.49 | 0.0392 |  |
| AQP4 | -0.73 | 0.0401 |  |
| CTD-2353F22.2 | -1.09 | 0.0401 |  |
| DHODH | -0.18 | 0.0401 |  |
| KREMEN1 | -0.29 | 0.0401 |  |
| LTBP3 | -0.28 | 0.0401 |  |
| PCDHGA3 | -0.72 | 0.0401 |  |
| SORCS2 | -0.42 | 0.0401 |  |
| SPPL2B | -0.21 | 0.0401 |  |
| ACOT11 | -0.37 | 0.0421 |  |
| ARHGAP5-AS1 | -0.37 | 0.0421 |  |
| TCF7 | -0.42 | 0.0422 |  |
| ADAMTSL5 | -0.46 | 0.0423 |  |
| NPFFR1 | -0.47 | 0.0423 |  |
| PHKG1 | -0.52 | 0.0423 |  |
| SLC14A1 | -1.03 | 0.0423 |  |
| TMCO4 | -0.34 | 0.0424 |  |
| BCL2 | -0.33 | 0.0436 |  |
| CRYL1 | -0.32 | 0.0436 |  |
| MAOB | -0.21 | 0.0436 |  |
| RHPN2 | -0.58 | 0.0436 |  |
| RP6-201G10.2 | -0.47 | 0.0446 |  |
| RP11-517I3.1 | -0.66 | 0.0447 |  |
| SLC4A11 | -0.53 | 0.0447 |  |
| ABCC11 | -0.50 | 0.0461 |  |
| PLXNB2 | -0.28 | 0.0468 |  |
| C10orf105 | -1.00 | 0.0468 |  |
| FUT10 | -0.43 | 0.0468 |  |
| GPT2 | -0.39 | 0.0468 |  |
| LCTL | -0.53 | 0.0468 |  |
| SLC34A3 | -0.49 | 0.0468 |  |
| SLC18B1 | -0.40 | 0.0479 |  |
| HHATL | -0.52 | 0.0479 |  |
| PRDM16 | -0.58 | 0.0483 |  |
| ATP13A4 | -0.67 | 0.0484 |  |
| HSPBAP1 | -0.21 | 0.0484 |  |
| MUC1 | -0.57 | 0.0484 |  |
| AC004019.13 | -0.58 | 0.0488 |  |
| AK4 | -0.26 | 0.0488 |  |
| BCAR3 | -0.38 | 0.0488 |  |
| EEF2K | -0.29 | 0.0488 |  |
| FOSB | -1.35 | 0.0488 |  |
| MROH7 | -0.39 | 0.0488 |  |
| NEBL | -0.28 | 0.0488 |  |
| PLCG1-AS1 | -0.54 | 0.0488 |  |
| PLEKHG4 | -0.36 | 0.0488 |  |
| RP11-106M7.1 | -0.40 | 0.0488 |  |
| RP11-1072A3.3 | -0.34 | 0.0488 |  |
| ZNF491 | -0.32 | 0.0488 |  |
| RP11-388C12.8 | -0.41 | 0.0489 |  |
| ADHFE1 | -0.44 | 0.0489 |  |
| ALS2CR12 | -0.28 | 0.0489 |  |
| GREB1 | -0.21 | 0.0489 |  |
| TPD52L1 | -0.53 | 0.0489 |  |
| VMAC | -0.24 | 0.0489 |  |
| CYP4F11 | -0.67 | 0.0494 |  |
| EPHX1 | -0.56 | 0.0496 |  |
| MTND1P23 | 3.26 | 1.20E-09 |  |
| MTCO1P12 | 2.38 | 3.87E-05 |  |
| ARPC5 | 0.22 | 0.0157 |  |
| SMIM10L1 | 0.26 | 0.0157 |  |
| ST8SIA2 | 0.76 | 0.0157 |  |
| RALA | 0.16 | 0.0176 |  |
| CASC15 | 0.27 | 0.0192 |  |
| ARPC2 | 0.22 | 0.0202 |  |
| BZW1 | 0.16 | 0.0202 |  |
| CSMD2 | 0.24 | 0.0202 |  |
| METTL9 | 0.16 | 0.0202 |  |
| SET | 0.17 | 0.0202 |  |
| NKAIN2 | 0.26 | 0.0210 |  |
| DR1 | 0.22 | 0.0234 |  |
| CELSR1 | 0.54 | 0.0242 |  |
| HSBP1 | 0.22 | 0.0259 |  |
| TMEM64 | 0.22 | 0.0268 |  |
| CHCHD3 | 0.15 | 0.0276 |  |
| RP11-271F18.4 | 0.33 | 0.0288 |  |
| SERBP1 | 0.14 | 0.0288 |  |
| LMO4 | 0.27 | 0.0291 |  |
| RP11-136K7.2 | 0.61 | 0.0291 |  |
| SOBP | 0.25 | 0.0291 |  |
| ANO4 | 0.26 | 0.0303 |  |
| CDH6 | 0.30 | 0.0310 |  |
| SDCBP | 0.14 | 0.0310 |  |
| H19 | 1.24 | 0.0329 |  |
| C8orf34 | 0.34 | 0.0330 |  |
| LGALSL | 0.21 | 0.0364 |  |
| PDCD2 | 0.15 | 0.0364 |  |
| USP12 | 0.27 | 0.0378 |  |
| PKP4 | 0.24 | 0.0384 |  |
| NDUFS4 | 0.23 | 0.0401 |  |
| SEMA3C | 0.37 | 0.0401 |  |
| UBXN2B | 0.21 | 0.0401 |  |
| NPPA | 0.60 | 0.0421 |  |
| RNF152 | 0.33 | 0.0421 |  |
| STMN2 | 0.38 | 0.0421 |  |
| ENPP6 | 0.47 | 0.0423 |  |
| SLC35G1 | 0.29 | 0.0423 |  |
| PTHLH | 0.36 | 0.0424 |  |
| SMARCE1 | 0.10 | 0.0436 |  |
| TC2N | 0.35 | 0.0436 |  |
| MEST | 0.24 | 0.0447 |  |
| SLN | 0.71 | 0.0447 |  |
| GNB4 | 0.25 | 0.0468 |  |
| GSG1 | 0.33 | 0.0468 |  |
| LINC00662 | 0.18 | 0.0468 |  |
| MARCH1 | 0.27 | 0.0468 |  |
| TCEB1 | 0.17 | 0.0479 |  |
| RAPGEF4 | 0.32 | 0.0483 |  |
| BLMH | 0.14 | 0.0484 |  |
| MAML3 | 0.24 | 0.0484 |  |
| MSANTD3-TMEFF1 | 0.24 | 0.0488 |  |
| NFU1 | 0.19 | 0.0488 |  |
| RP11-1263C18.1 | 0.24 | 0.0488 |  |
| YWHAB | 0.22 | 0.0488 |  |
| CXADR | 0.24 | 0.0489 |  |
| CDC42 | 0.19 | 0.0489 |  |
| MTPN | 0.21 | 0.0489 |  |
| EGFEM1P | 0.40 | 0.0494 |  |
| PPEF1 | 0.38 | 0.0494 |  |
| TYRP1 | 0.49 | 0.0496 |  |
| ZC3H15 | 0.21 | 0.0500 |  |
| **Table S4B** |  |  |  |
| Longevity in subg-ACC (Mood Disorders & Controls) | | | |
| GeneName | log2FoldChange | q-adjusted |  |
| ARHGAP10 | -0.33 | 0.0215 |  |
| CPXM1 | -0.58 | 0.0362 |  |
| PTGER4 | -0.45 | 0.0362 |  |
| RANBP17 | -0.21 | 0.0362 |  |
| ADGRG6 | -0.38 | 0.0426 |  |
| TNFRSF10A | -0.45 | 0.0426 |  |
| ISG15 | -0.36 | 0.0435 |  |
| MALL | -0.34 | 0.0435 |  |
| RNF152 | -0.25 | 0.0435 |  |
| SOX4 | -0.22 | 0.0435 |  |
| TH | -0.83 | 0.0435 |  |
| ZFP37 | -0.15 | 0.0435 |  |
| ACCS | 0.20 | 0.0435 |  |
| ROCK1P1 | 0.76 | 0.0435 |  |
| Longevity in subg-ACC (Mood Disorders only) | | |  |
| GeneName | log2FoldChange | q-adjusted |  |
| ARHGAP10 | -0.45 | 0.0037 |  |
| AC012146.7 | -0.35 | 0.0164 |  |
| FBXO18 | -0.11 | 0.0240 |  |
| RACGAP1 | -0.18 | 0.0240 |  |
| USP12 | -0.17 | 0.0240 |  |
| COL11A1 | -0.22 | 0.0400 |  |
| FAM184B | -0.20 | 0.0400 |  |
| WWC2 | -0.17 | 0.0400 |  |
| ZNF23 | -0.13 | 0.0400 |  |
| B4GALT2 | -0.26 | 0.0485 |  |
| ROCK1P1 | 1.22 | 0.0014 |  |
| RP11-474N24.6 | 0.32 | 0.0164 |  |
| CTC-591M7.1 | 0.25 | 0.0240 |  |
| PRELP | 0.37 | 0.0240 |  |
| RP11-138A9.1 | 0.32 | 0.0240 |  |
| RP11-138A9.2 | 0.26 | 0.0240 |  |
| RP11-269G24.7 | 0.47 | 0.0386 |  |
| C12orf60 | 0.17 | 0.0400 |  |
| CTD-2014D20.1 | 0.35 | 0.0400 |  |
| RP11-265O12.1 | 0.27 | 0.0400 |  |
| RP11-214K3.21 | 0.26 | 0.0485 |  |
| RP11-283I3.6 | 0.18 | 0.0485 |  |

| **Table S5**  **Table S5A** | | |
| --- | --- | --- |
| Longevity in Ant-Ins (Controls Only) | | |
| GeneName | log2FoldChange | q-adjusted |
| RP11-638I2.6 | -1.24 | 8.03E-06 |
| RP4-738P15.6 | -1.39 | 0.0019 |
| CTB-43P18.1 | -0.64 | 0.0030 |
| HCG25 | -0.82 | 0.0030 |
| RP1-257A7.5 | -0.69 | 0.0030 |
| RP11-215P8.2 | -0.60 | 0.0031 |
| PSMA2 | -0.23 | 0.0032 |
| RPL9 | -0.89 | 0.0044 |
| PET100 | -0.47 | 0.0081 |
| COL6A3 | -1.05 | 0.0084 |
| MT-TS1 | -1.10 | 0.0084 |
| RP11-603J24.17 | -0.93 | 0.0102 |
| H2BFM | -0.59 | 0.0137 |
| C19orf81 | -0.58 | 0.0148 |
| RPS29 | -0.37 | 0.0256 |
| AC005944.2 | -0.71 | 0.0262 |
| SLIRP | -0.35 | 0.0278 |
| AC009133.12 | -0.53 | 0.0323 |
| ATP5I | -0.49 | 0.0349 |
| AC007192.6 | -1.45 | 0.0388 |
| CTC-490E21.14 | -0.59 | 0.0395 |
| C10orf67 | -0.36 | 0.0401 |
| CTD-2562J17.7 | -1.06 | 0.0454 |
| RP11-361L15.3 | -0.41 | 0.0454 |
| GSG1 | -0.48 | 0.0486 |
| FAM60DP | 3.26 | 3.64E-06 |
| RP11-325O24.5 | 3.82 | 3.64E-06 |
| RPSAP48 | 3.14 | 1.33E-05 |
| PSG2 | 3.02 | 3.81E-05 |
| FGF23 | 2.44 | 5.31E-05 |
| LPAR4 | 2.56 | 0.0002 |
| S1PR2 | 1.50 | 0.0003 |
| RP11-44M6.3 | 2.60 | 0.0003 |
| MIR3648-1 | 0.63 | 0.0005 |
| MIR3648-2 | 0.63 | 0.0005 |
| NPM1P40 | 1.99 | 0.0005 |
| RP11-715J22.2 | 1.78 | 0.0005 |
| SHOX | 1.60 | 0.0005 |
| RP11-38O23.4 | 2.03 | 0.0006 |
| AC009404.2 | 1.91 | 0.0015 |
| FOXP4 | 0.31 | 0.0019 |
| WT1-AS | 2.16 | 0.0019 |
| RP11-87N24.3 | 1.93 | 0.0026 |
| MIR663AHG | 0.58 | 0.0027 |
| LRRC69 | 1.54 | 0.0030 |
| LINC00273 | 0.54 | 0.0031 |
| BMP8B | 0.59 | 0.0048 |
| RP1-17K7.2 | 1.31 | 0.0075 |
| ZNF302 | 0.68 | 0.0137 |
| MIR6087 | 0.86 | 0.0152 |
| RP11-217O12.1 | 1.53 | 0.0177 |
| CTD-2527I21.4 | 0.64 | 0.0211 |
| ZNF121 | 0.38 | 0.0227 |
| MIR3687-1 | 0.43 | 0.0256 |
| MIR3687-2 | 0.43 | 0.0256 |
| CTC-204F22.1 | 0.70 | 0.0298 |
| AGAP7P | 1.39 | 0.0405 |
| SYNDIG1L | 1.74 | 0.0447 |
| NTN3 | 0.49 | 0.0494 |
| **Table S5B** |  |  |
| Longevity in Subg-ACC (Controls Only) | | |

| Gene Name | Log2 Fold Change | q-adjusted |
| --- | --- | --- |
| SELE | -2.086484395 | 0.00272583 |
| TNFRSF10A | -0.755720757 | 0.00272583 |
| TMEM45B | -0.356020359 | 0.0053495 |
| SEMA3F | -0.59504703 | 0.00898067 |
| ADGRL4 | -0.45364579 | 0.01710602 |
| PUDP | -0.342556881 | 0.01971902 |
| VASP | -0.369964092 | 0.03062816 |
| PTGER4 | -0.695458526 | 0.03530824 |
| HSPA1A | -1.485909733 | 0.0381877 |
| HSPB1 | -1.179515039 | 0.0381877 |
| PLA1A | -1.042718997 | 0.0381877 |
| PLEKHG1 | -0.288379801 | 0.0381877 |
| SOCS3 | -1.618596536 | 0.0464215 |
| ADAMTS1 | -0.959752544 | 0.04833743 |
| DNAJB1 | -1.126933559 | 0.04876992 |
| ICAM2 | -0.393988889 | 0.04876992 |
| NOS3 | -0.495096445 | 0.04876992 |
| ORAI1 | -0.338937288 | 0.04876992 |
| AF131216.6 | -0.205331634 | 0.04887092 |
| CNN2 | -0.45579366 | 0.04887092 |
| STX16-NPEPL1 | 0.395075302 | 0.04887092 |

| **Table S6** | | |
| --- | --- | --- |
| **Table S6A.** | | |
| Ant-Ins Lowest 20 vs. Highest 20 Psychiatric Morbidity  (Mood Disorders & Controls) | | |
| GeneName | Log2 Fold Change | q-adjusted |
| NPAS4 | -0.12 | 0.02 |
| **Table S6B**. | | |
| Ant-Ins Lowest 20 vs. Highest 20 Psychiatric Morbidity  (Mood Disorders Only) | | |
| GeneName | Log2 Fold Change | q-adjusted |
| No Gene | NS | NS |

| **Table S7** | | |
| --- | --- | --- |
| **Table S7A** | | |
| sgACC Lowest 20 vs. Highest 20 Psychiatric Morbidity (Mood Disorders & Controls) | | |
| GeneName | log2FoldChange | q-adjusted |
| RP1-167G20.1 | -0.877002039 | 0.00452634 |
| RP11-98D18.15 | -0.724926784 | 0.02728451 |
| SNORD113-2 | -0.587366678 | 0.04990453 |
| LAMB3 | -0.444975015 | 0.04653281 |
| C1orf132 | -0.364458633 | 0.03463994 |
| STARD8 | 0.29902623 | 0.04864675 |
| TTC32 | 0.330729284 | 0.045337 |
| FPGT-TNNI3K | 0.411101495 | 0.03670549 |
| TMEM187 | 0.430865111 | 0.02728451 |
| SLC2A4 | 0.455605566 | 0.03670549 |
| VWF | 0.599546082 | 0.04990453 |
| KANK3 | 0.604297074 | 0.01055268 |
| FAM101B | 0.668751171 | 0.04653281 |
| CLDN5 | 0.702514793 | 0.04653281 |
| FLT1 | 0.704786865 | 0.02728451 |
| ADAMTS9-AS1 | 0.741030174 | 0.03670549 |
| ATHL1 | 0.91922823 | 0.04908813 |
| TGFB3 | 0.965307862 | 0.04990453 |
| LRRC32 | 0.96851777 | 0.03124542 |
| TM4SF1 | 1.003397954 | 0.03670549 |
| DDIT4 | 1.040727052 | 0.04990453 |
| CDK2 | 1.099152007 | 0.03670549 |
| IFITM3 | 1.10533146 | 0.04908813 |
| ADAMTS9 | 1.150835433 | 0.03334998 |
| IFITM1 | 1.191864607 | 0.00543532 |
| OSMR | 1.254295112 | 0.04653281 |
| MT1M | 1.284933497 | 0.01055268 |
| IL1R1 | 1.285525005 | 0.04990453 |
| HAMP | 1.290936248 | 0.04908813 |
| KIAA0040 | 1.310680312 | 0.04653281 |
| C10orf10 | 1.548180748 | 0.00378147 |
| ANGPTL4 | 1.560992237 | 0.03463994 |
| STON1-GTF2A1L | 1.599711214 | 0.04908813 |
| MT1X | 1.604506037 | 0.01187363 |
| IFITM2 | 1.681454271 | 0.00441438 |
| SFN | 2.086497044 | 0.03664745 |
| HILPDA | 2.162150261 | 0.00937067 |
| ADM | 2.411503273 | 0.00937067 |
| **Table S7B** | | |
| sgACC Lowest 20 vs Highest 20 Psychiatric Morbidity (Mood Disorders Only) | | |
| GeneName | log2FoldChange | q-adjusted |
| MTND2P28 | -1.474105007 | 0.01310846 |
| ARC | -0.978329496 | 0.02169186 |
| RP13-870H17.3 | -0.844750279 | 0.01707175 |
| RP11-867G23.10 | -0.837706677 | 0.02281121 |
| RP11-52L5.6 | -0.69764366 | 0.02364908 |
| CACNA1G-AS1 | -0.668752739 | 0.01854167 |
| RP1-167G20.1 | -0.602284494 | 0.03793296 |
| DUSP27 | -0.568993179 | 0.04878035 |
| CDC20P1 | -0.540684716 | 0.02889881 |
| F7 | -0.539476544 | 0.00486875 |
| SNORD113-2 | -0.506426759 | 0.01782209 |
| CTB-58E17.5 | -0.491602292 | 0.04391114 |
| RTL1 | -0.482276895 | 0.041726 |
| AC009495.3 | -0.467746838 | 0.04137867 |
| LINC00996 | -0.460621848 | 0.01593218 |
| RP4-778K6.3 | -0.449182168 | 0.03252294 |
| RP4-569M23.4 | -0.416667541 | 0.01746233 |
| LAMB3 | -0.393436452 | 0.02256006 |
